# Hotspots and frontiers of the relationship between gastric cancer and cancer-associated fibroblasts: a bibliometric analysis

**DOI:** 10.3389/fonc.2025.1576696

**Published:** 2025-05-23

**Authors:** Xiangcheng Hu, Lingling Ren, Shuyuan Zhu, Haiping Shen, Chengyong Qian, Guanglan Chen, Fangling Chen

**Affiliations:** Digestive Endoscopy Center, The Second People’s Hospital of Lishui, Lishui, China

**Keywords:** cancer-associated fibroblasts, gastric cancer, bibliometrics, tumor microenvironment, hot spot

## Abstract

**Background:**

Cancer-Associated Fibroblasts (CAFs) are pivotal stromal constituents within the Tumor Microenvironment (TME), characterized by marked heterogeneity and plasticity. Over the past two decades, a notable association between Gastric Cancer (GC) and CAFs has been established. Despite this, there remains a paucity of comprehensive data to guide researchers in understanding the prevalence and potential research trajectories concerning GC and CAFs.

**Methods:**

This study conducted an extensive literature search within the Web of Science Core Collection database from January 1, 2003, to December 31, 2023. Bibliometric analysis and visualization were performed using VOSviewer, CiteSpace, R software, and Microsoft Excel.

**Results:**

A total of 1170 articles were included. These articles were disseminated across 200 journals and incorporated 1800 distinct keywords. A notable surge in publications has been observed from 2011 to 2023. China emerged as the leading contributor to both article count and citations. Prominent research institutions in this domain include Osaka City University, Shanghai Jiaotong University, and National Cancer Research Center Hospital. Notable researchers, such as Masakazu Yashiro and Kosei Hirakawa from Osaka City University and Zhenggang Zhu from Shanghai Jiaotong University, were among the most productive and highly cited authors. FRONTIERS IN ONCOLOGY boasts the highest number of publications, whereas ONCOGENE ranks as the most cited journal. The primary research foci within the realm of CAFs and GC encompass the impact of CAFs on GC cell proliferation, angiogenesis, invasion, metastasis, epithelial-mesenchymal transition, immunosuppression, drug resistance, and the interplay between CAFs and GC.

**Conclusion:**

Using bibliometric analysis, this study presents a panoramic view of the research landscape of CAFs and GC from 2003 to 2023. It highlights prominent research areas and anticipates future directions with the aim of offering valuable insights and strategic recommendations for future endeavors in this field.

## 1 Introduction

Gastric Cancer (GC) ranks among the most common malignancies globally. It is estimated that there are one million new cases of gastric cancer each year, making it the fifth most diagnosed cancer worldwide. Due to its frequent late-stage diagnosis, gastric cancer exhibits a high mortality rate and is the third leading cause of cancer-related deaths (1). Currently, the main treatment methods for GC include radical resection, neoadjuvant therapy, and chemotherapy (2). However, the prognosis of gastric cancer patients is still not ideal, which suggests that our current understanding of gastric cancer is insufficient, and we still need to increase the exploration of unknown areas of gastric cancer in the future.

Fibroblasts, prevalent in the extracellular matrix of human tissues, are crucial in wound healing, tissue inflammation, and organ fibrosis (3). Recent studies indicate that fibroblasts within tumor tissues can transform into cancer-associated fibroblasts (CAFs) upon receiving stimulatory signals. CAFs secrete a variety of cytokines and metabolites that inhibit the function of immune cells and promote the progression, invasion, angiogenesis, and drug resistance of tumor cells (4–6). Therefore, CAFs have become the focus of current research. However, most current research on CAFs focuses on breast, pancreatic, and colorectal cancers (7). In recent years, an increasing number of researchers have begun to pay attention to the effect of CAFs on the occurrence and development of gastric cancer and have explored the mechanism, which has become a new research trend in GC and CAFs.

As early as 1969, Pritchard proposed the concept of bibliometrics, which is defined as “the application of mathematical and statistical methods to the calculation and analysis of different aspects of text information to reveal the process of text information and the nature and trend of discipline development” (8). In recent years, bibliometrics has been widely used to explore the characteristics of academic publications in specific fields of research, including influential countries, journals, institutions, authors, favorable publications, references, and keywords (9). Bibliometrics uses several analytical tools to generate graphs to visualize collaboration between countries, institutions, and authors. In addition, it allows researchers to quickly grasp the evolution and frontiers of specific research areas (10). As basic research on CAFs in GC continues to increase, bibliometric analysis in this field may provide direction to investigate clinical questions and improve the awareness of research trends. Therefore, a bibliometric analysis of the role of CAFs in GC is required. Through this analysis, the research status, hotspots, and future research trends of CAFs in the field of GC disease can be determined.

In this study, we aimed to analyze the relationship between GC and CAFs through bibliometrics to help experts and newcomers broaden the breadth of their field, find new topics of interest, and specify future research plans in a visual manner.

## 2 Materials and methods

### 2.1 Database and search strategy

We utilized the Web of Science Core Collection (WOSCC) for literature retrieval. To enhance the credibility and precision of our findings, we employed ‘cancer-associated fibroblasts’ and ‘gastric cancer’ as search terms, refining our search strategy with insights from prior research (11). A detailed search formula is provided in the **Supplementary Material**. The release period spanned 20 years, beginning on January 1, 2003, and ending on December 31, 2023. The articles included in this study were classified as articles or review articles and were written in English. To mitigate the potential for systematic bias due to database updates, we conducted a comprehensive search and screening of publications, which was completed on October 04, 2024.

### 2.2 Data analysis

We created flow charts in Microsoft Word 2019, and statistical tables and fitted curve analyses in Microsoft Excel 2019. Lotka’s law analysis and Bradford’s law analysis were performed using the bibliometrix 4.1.3 tool in R 4.3.1. Through the online bibliometrics website (https://bibliometric.com/), we were able to visualize international collaborations between countries, and Origin 2024 was used to perform national visualization analysis. In addition, VOSviewer 1.6.19 and CiteSpace 6.2R4 were used for the bibliometric analysis of institutions, authors, journals, and keywords. Our primary aim was to analyze co-authorship, co-occurrence, and co-citation patterns. We consolidated overlapping items, corrected spelling errors manually, and conducted data cleaning prior to exporting for further analysis.

### 2.3 Lotka’s law and Bradford’s law

Lotka’s law, which is based on a power-law distribution, describes the relationship between an author’s productivity and publication frequency (12). The number of authors who have published a single article is significantly higher than the number of authors who have published multiple articles. By analyzing the distribution of publications, Lotka’s law can quantify the productivity of authors and assess the concentration or inequality within a field. Lotka’s law is mathematically expressed as follows (13):


$$A\left(n\right)=A\left(1\right)/n^{2}$$

In the equation, A(n) represents the number of authors who have published n papers, while A (1) denotes those who have published only one paper.

Bradford’s law was employed to assess the productivity of scientific journals, categorizing them into three distinct zones. Zone 1, the core, includes the most prolific journals. Zone 2, the relevant zone, comprises 25% of the total journal count. Zone 3, the irrelevant zone, consists of journals that have infrequently published on the topic (14).

## 3 Result

**Figure 1:**
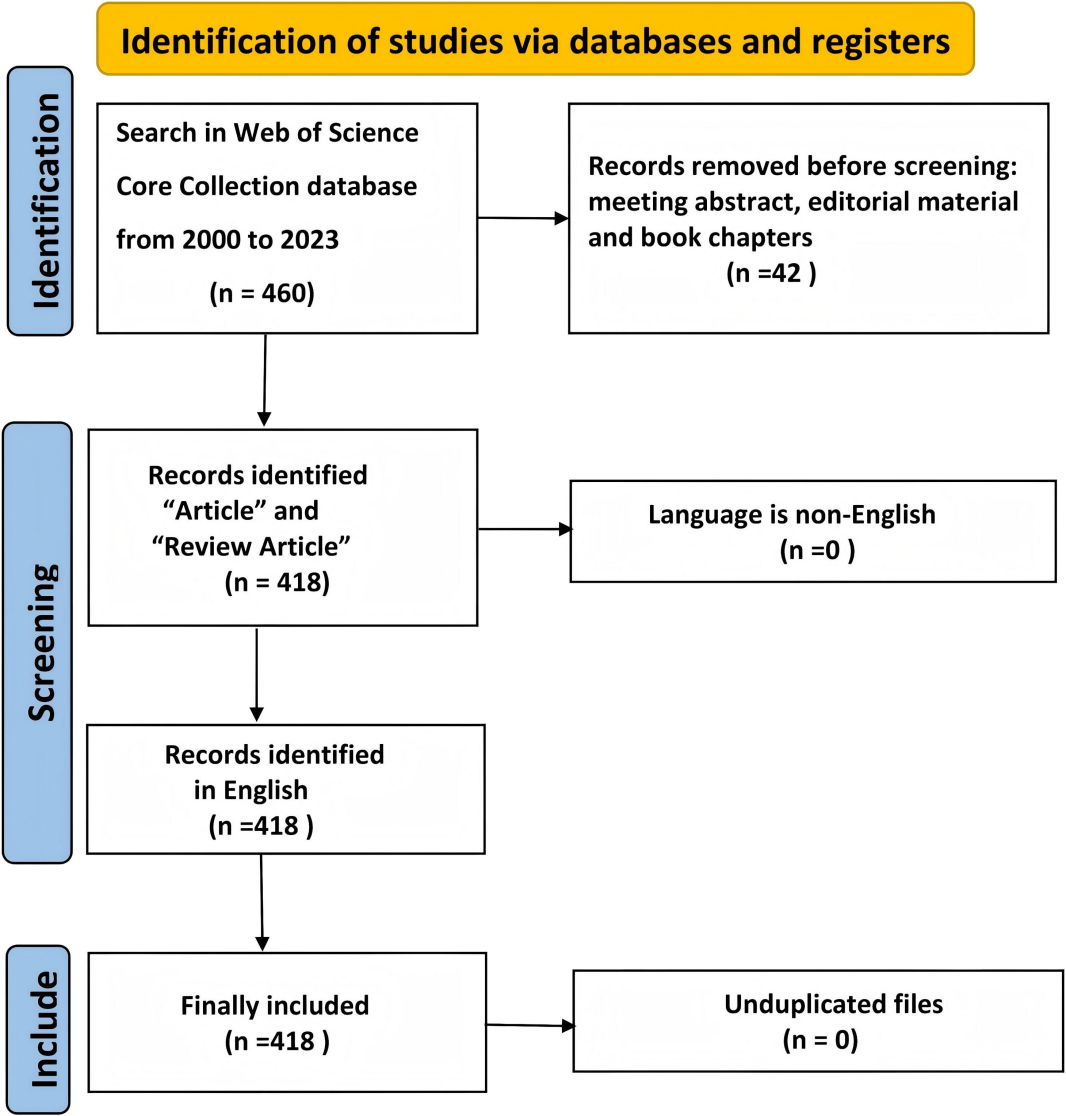
Flowchart of the search strategy and exclusion criteria.

We retrieved 460 papers from WOSCC, applying exclusion criteria based on publication date, article type, and language, ultimately selecting 418 papers for bibliometric analysis. A flow chart is shown in **Figure 1**.

### 3.1 Analysis of the number of publications

**Figure 2:**
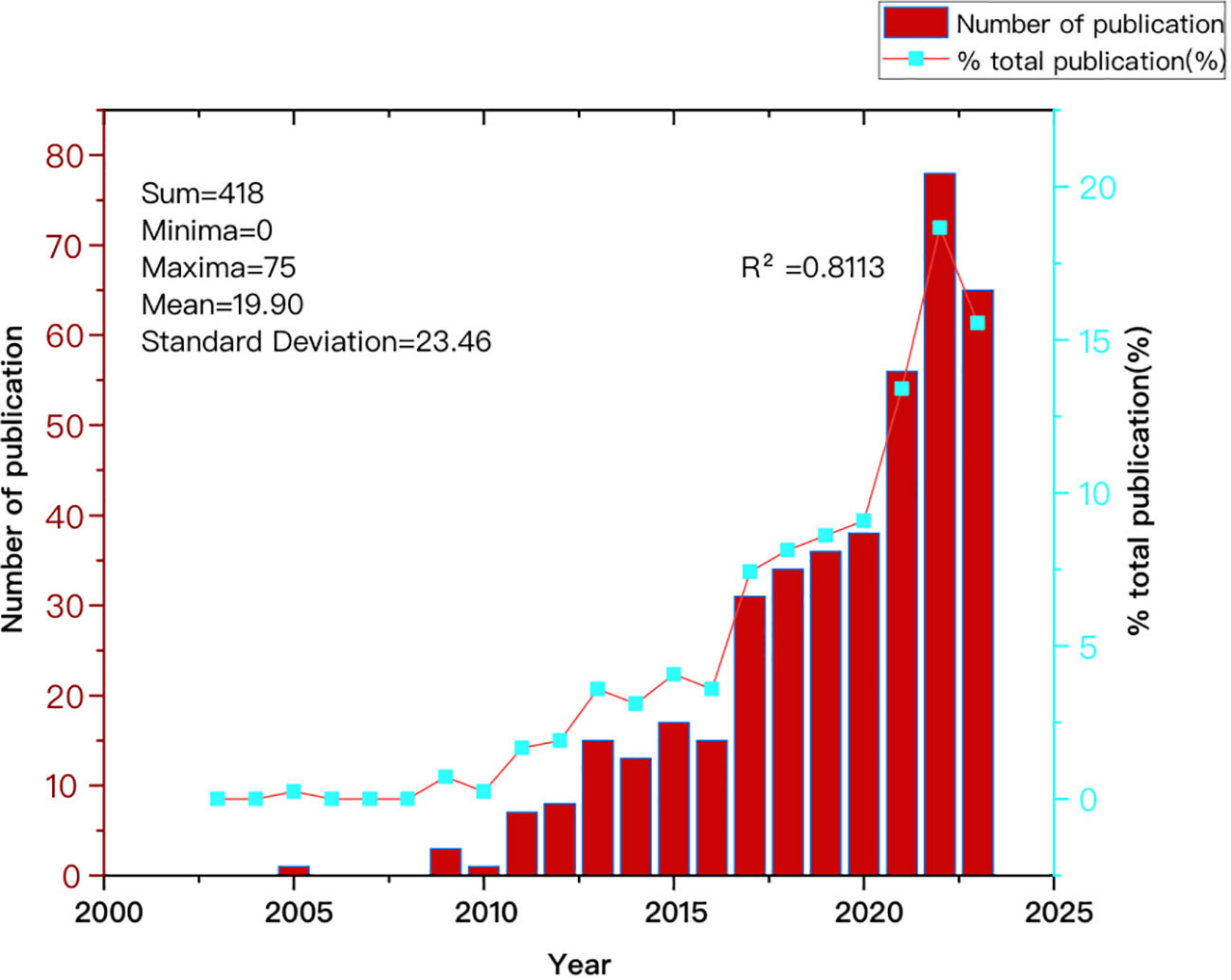
Trend graph depicting the annual growth in publications on cancer-associated fibroblasts and gastric cancer.

**Figure 2** illustrates the annual growth trend in articles published by CAFs and GC, demonstrating a general year-on-year increase from 2003 to 2023. The number of articles produced from 2003 to 2010 was small, with a total of five articles. Starting in 2011, the number of published articles rose steadily, reaching a cumulative total of 413 by 2023. This figure represents approximately 98.8% of the total number of articles published in the broader period from 2003 to 2023. Between 2017 and 2023, the number of publications increased significantly and rapidly, with more than 31 publications in 2019 and more than 60 publications in 2023, although the output was slightly lower than that in 2022. This suggests that the nuanced relationship between CAFs and GC is garnering increased attention, emerging as a prominent and rapidly developing research focus.

### 3.2 Situation of countries/regions and institutions

**Figure 3:**
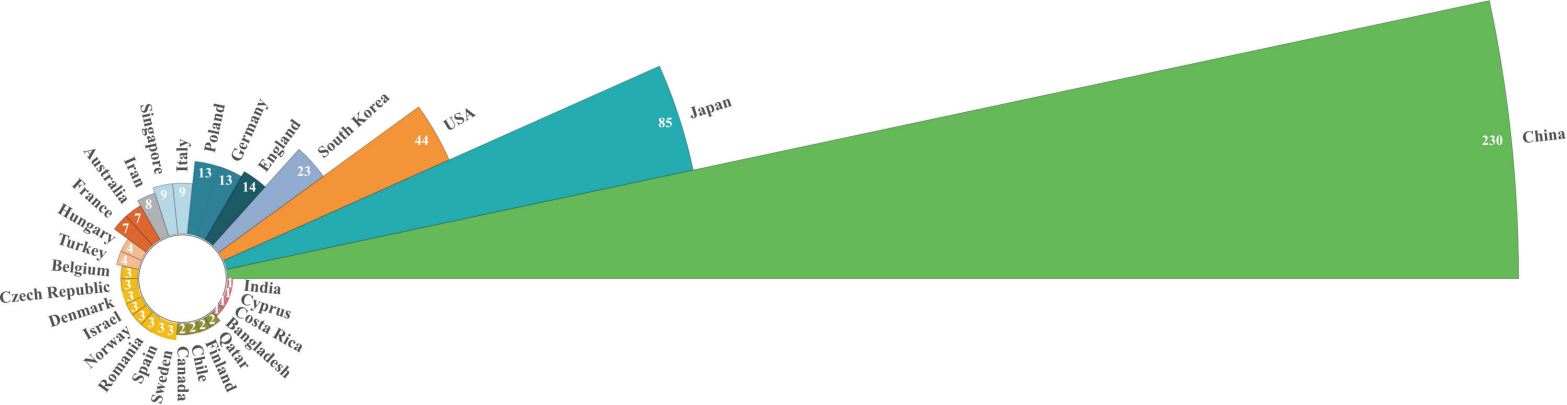
Nightingale’s Rose chart of the top 30 countries by publications.

**Figure 4:**
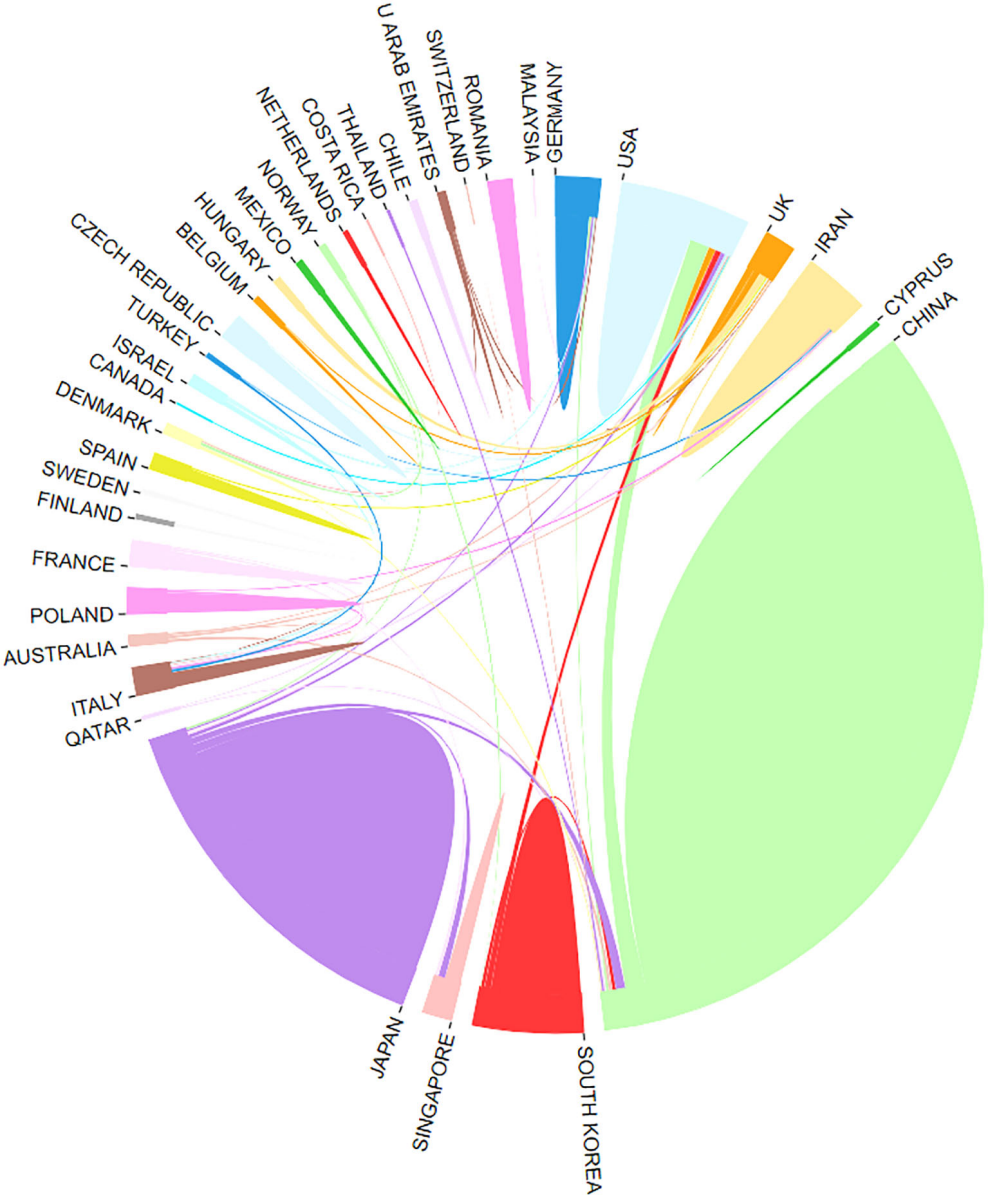
Network of cooperation between countries.

Publications on CAFs and GC have emerged from 39 countries. **Figure 3** shows the top 30 countries in terms of productivity. China had the largest number of publications (N=230), followed by Japan (N=85) and the United States (N=44). China was cited most frequently (N=7742), followed by Japan (N=3573) and the United States (N=3208). China leads in both publication and citation counts, reflecting the significant academic influence and high quality, innovation, and recognition of Chinese research in this field. **Figure 4** illustrates strong collaboration between China, the United States, Japan, and South Korea, suggesting that South Korea is poised for substantial advancements in the future.

**Figure 5:**
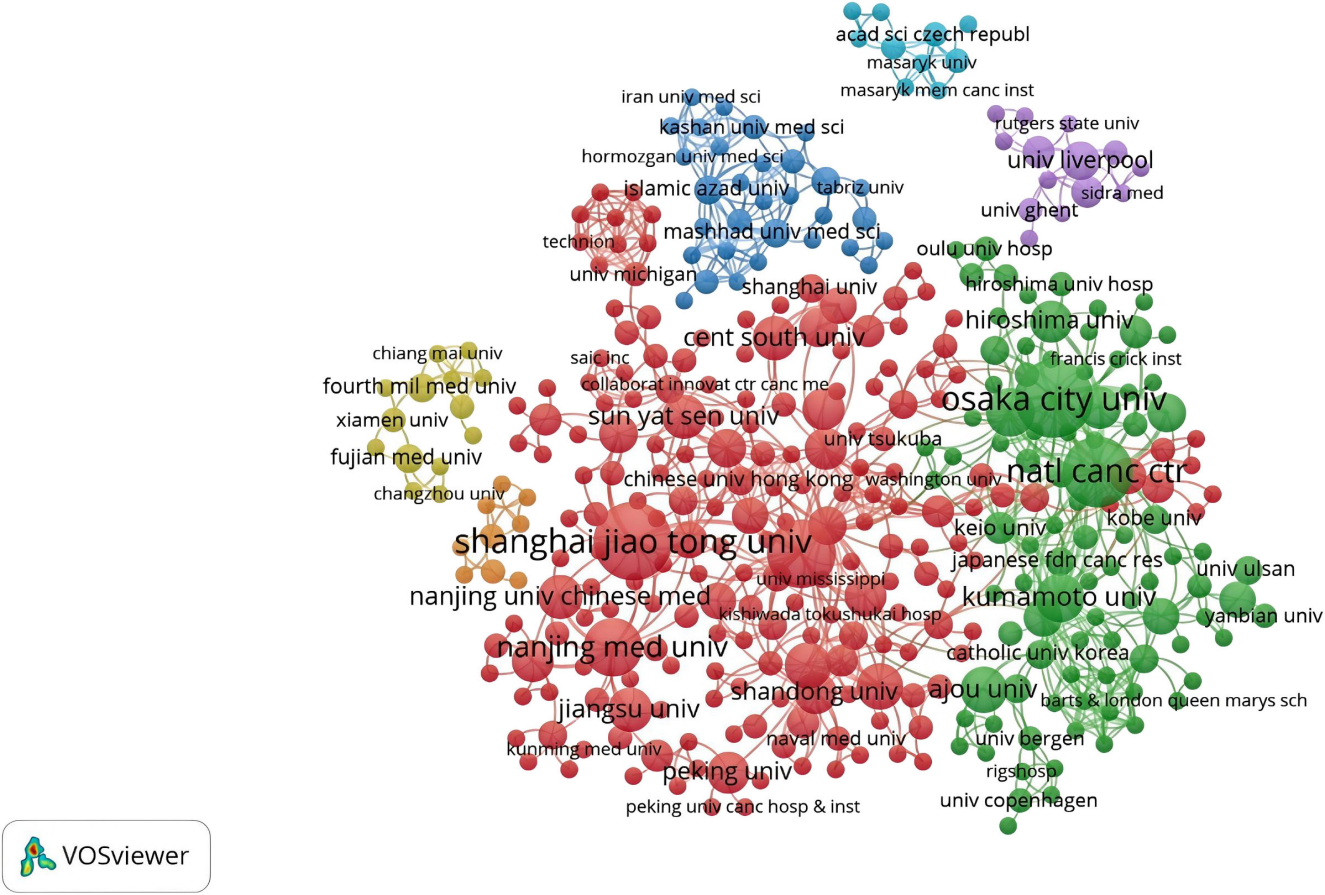
A network visualization map of institutions in the field of cancer-associated fibroblasts and gastric cancer.

There were 555 institutions in the seven main clusters (**Figure 5**). The institutions with the largest number of articles were Osaka City University, Shanghai Jiao Tong University, and the National Cancer Center Hospital, with 29, 29, and 23 articles, respectively. Moreover, half of the top ten institutions is based in China, underscoring the significant engagement of Chinese institutions in CAFs and GC research and highlighting their prominent role and contributions in this field. The National Cancer Center Hospital (N=1445) was the most frequently cited, followed by Osaka City University (N=1420), Shanghai Jiao Tong University (N=1390), Nanjing Medical University (N=665), and Kumamoto University (N=621). All of them were from China and Japan, indicating that China and Japan had an important voice in this field. However, it also shows that international agencies are not working closely enough.

### 3.3 Authors analysis

**Figure 6:**
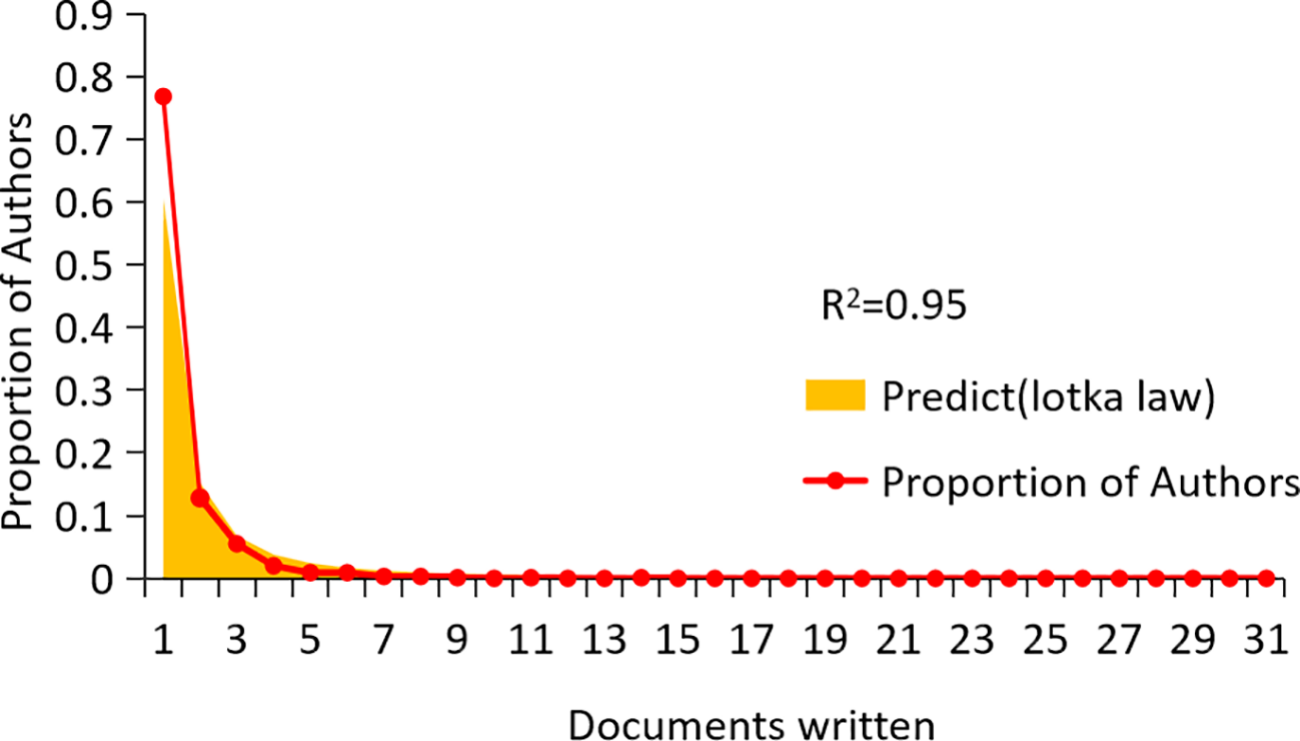
Scientific productivity of authors based on Lotka’ slaw.

**Figure 7:**
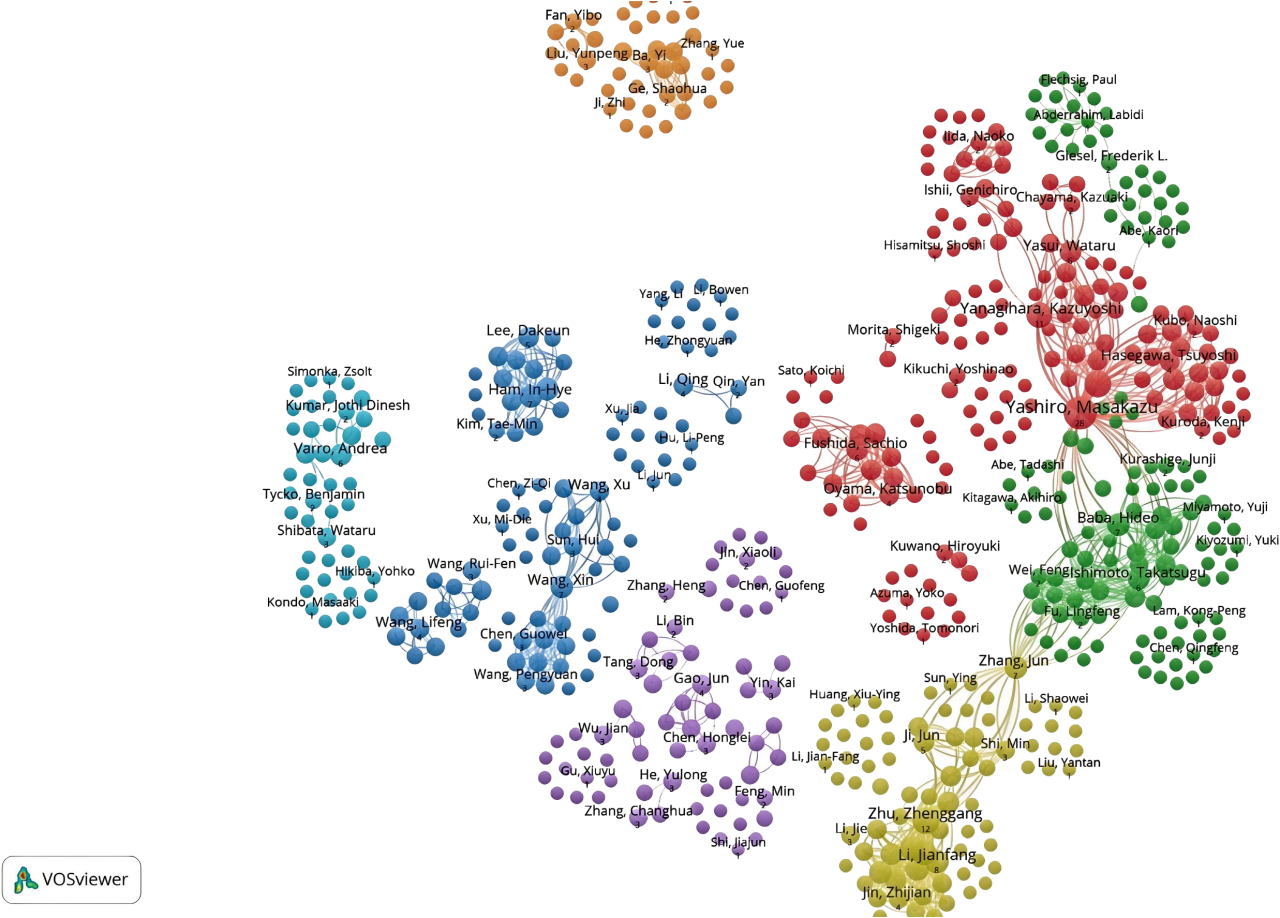
Network diagram of author collaborations for cancer-associated fibroblasts and gastric cancer studies.

A total of 2,358 authors contributed to CAFs and GC research. **Figure 6** indicates a high degree of consistency (R²=0.95) between the actual data and the predictions of Lotka’s Law, reflecting that author productivity aligns with this law’s characteristics. Calculations using the bibliometrix 4.1.3 package show that 76.8% of researchers only publish one paper, 12.8% publish two, and a mere 5.5% publish three. As the number of papers increases, the proportion of researchers decreases rapidly. This mirrors the common academic scenario where a small group of highly productive researchers contribute most of the output, while the majority have relatively lower output. These highly productive researchers are usually key figures in their fields. **Table 1** presents the top 20 authors distinguished by high productivity and citation rates in this domain. The top three authors were all from Japan and China, with Masakazu Yashiro (N=31) from Osaka City University publishing the most papers. This was followed by Kosei Hirakawa from Osaka City University (N=14) and Zhenggang Zhu from Shanghai Jiao Tong University (N=14), who published many papers. At the same time, the most cited authors are the three authors. It can be seen that they have made significant contributions to the study of CAFs and GC and are leaders in this field. Additionally, Bingya Liu and Liping Su. (11 publications, 118 citations) from Shanghai Jiao Tong University and Jianfang Li from the same hospital (10 publications, 117 citations) are also important researchers in the field of CAFs. Our analysis identified positive collaboration among the seven distinct author clusters (**Figure 7**). Notably, there is a degree of interaction between interconnected nodes across different clusters, specifically involving Masakazu Yashiro, Xin Wang, Andrea Varro, Hideo Baba, and Zhenggang Zhu.

**Table 1:** The top 20 most prolific and cited authors in the field of cancer-associated fibroblasts and gastric cancer.

Rank	Authors	Counts	Total citations	Average citation	Number of first author	Number of corresponding authors
1	Yashiro, M	31	231	7.45	2	10
2	Hirakawa, K	14	159	11.36	0	0
3	Zhu, ZG	14	134	9.57	0	1
4	Wang, X	13	54	4.15	0	1
5	Yanagihara, K	12	45	3.75	0	0
6	Liu, BY	11	118	10.73	0	3
7	Su, LP	11	118	10.73	0	3
8	Zhang, J	11	24	2.18	2	4
9	Li, JF	10	117	11.7	0	0
10	Wu, XY	9	85	9.44	2	0
11	Baba, H	9	80	8.89	0	1
12	Li, C	9	59	6.56	0	1
13	Ishimoto, T	8	67	8.38	1	0
14	Wang, LF	8	60	7.5	0	4
15	Lee, D	8	50	6.25	1	2
16	Tanaka, M	8	41	5.13	2	6
17	Zhang, H	8	33	4.13	1	0
18	Zhang, X	8	33	4.13	0	2
19	Wang, Y	8	18	2.25	1	0
20	Yu, BQ	7	54	7.71	1	0

### 3.4 Journals analysis

**Figure 8:**
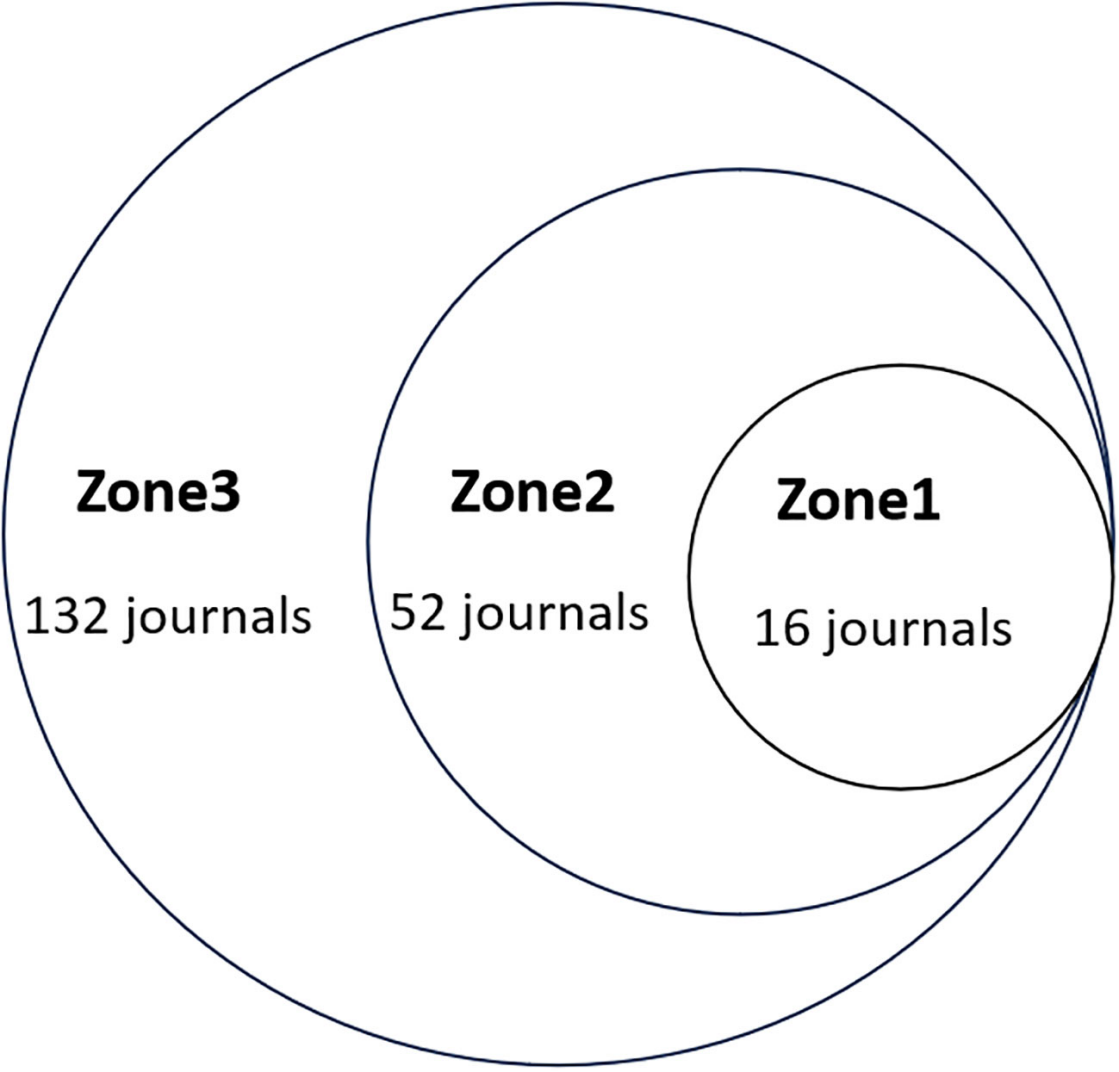
Scientific productivity of journals based on Bradford’s law.

**Figure 9:**
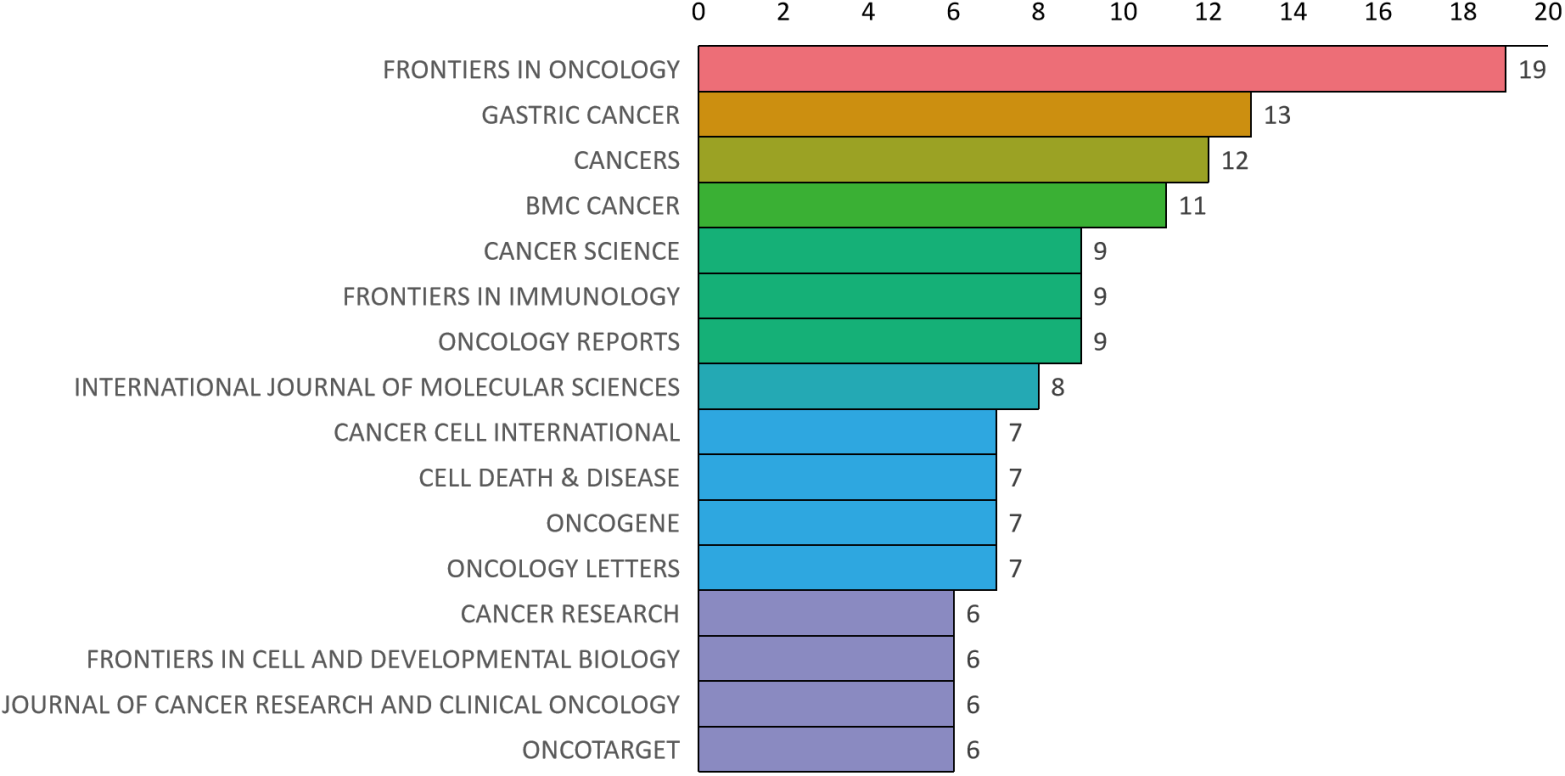
Distribution map of journal publications in zone one.

**Figure 10:**
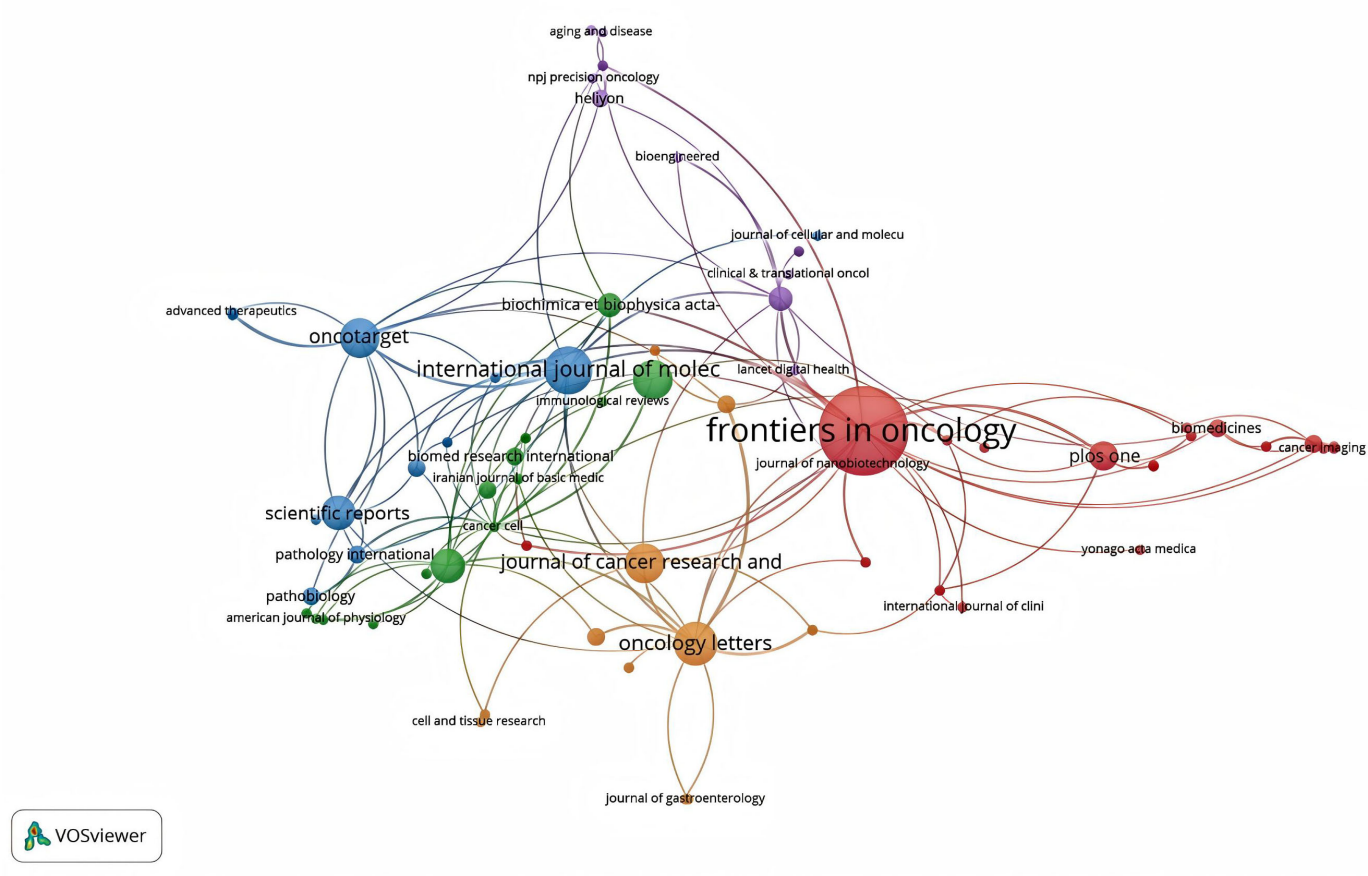
The network diagram of cited journals.

Bradford’s law was utilized to assess the representation of CAFs and GC in leading journals (15). As shown in **Figure 8**, the literature sources are divided into three zones: 16 journals in Zone 1, 52 in Zone 2, and 132 in Zone 3. This reveals that most literature in this field is concentrated in a few core journals (Zone 1). As we move outward from Zone 1 to Zone 3, the number of journals increases, but each journal contributes significantly fewer papers. This means that a small number of core journals publish most of the important papers, while many other journals only publish a few relevant papers. This distribution aligns with the typical pattern described by Bradford’s Law. **Figure 9** shows the top 16 core journals in the field, with the top five being FRONTIERS IN ONCOLOGY (N=19), GASTRIC CANCER (N=13), CANCERS (N=12), BMC CANCER (N=11), and CANCER SCIENCE (N=9). **Table 2** highlights that the top 16 journals by publication volume comprise 10 with Q1 Journal Citation Reports (JCR) rankings, three with Q2 rankings, and six with an impact factor exceeding 5. ONCOGENE was the most cited journal among the top 10 cited journals (N=68), followed by CANCER SCIENCE (N=59), and CANCER RESEARCH (N=44). There were 10 journals with Q1 JCR zoning and 3 journals with Q2 JCR zoning. This suggests that these journals are valued most by scholars and best reflect the current state of research. **Figure 10** presents the cited journal network, illustrating inter-journal associations. The network is divided into five clusters, where node size reflects co-citation counts and connection thickness indicates association strength. Journals sharing the same color focus on similar topics, predominantly within red clusters centered on cancer research, highlighting the strongest thematic connections and most frequent citations.

**Table 2:** The top 16 issued and cited journals for cancer-associated fibroblasts and gastric cancer studies base zone one.

Rank	Journal title	Records	Total citations	Average citation	JCR (2024)	IF (2024)
1	FRONTIERS IN ONCOLOGY	19	14	0.74	Q2	3.5
2	GASTRIC CANCER	13	36	2.77	Q1	6
3	CANCERS	12	40	3.33	Q1	4.5
4	BMC CANCER	11	19	1.73	Q2	3.4
5	CANCER SCIENCE	9	59	6.56	Q1	4.5
6	ONCOLOGY REPORTS	9	22	2.44	Q2	3.8
7	FRONTIERS IN IMMUNOLOGY	9	4	0.44	Q1	5.7
8	INTERNATIONAL JOURNAL OF MOLECULAR SCIENCES	8	10	1.25	Q1	4.9
9	ONCOGENE	7	68	9.71	Q1	6.9
10	CELL DEATH & DISEASE	7	32	4.57	Q1	8.1
11	ONCOLOGY LETTERS	7	22	3.14	Q3	2.5
12	CANCER CELL INTERNATIONAL	7	20	2.86	Q1	5.3
13	CANCER RESEARCH	6	44	7.33	Q1	12.5
14	ONCOTARGET	6	40	6.67	/	0
15	JOURNAL OF CANCER RESEARCH AND CLINICAL ONCOLOGY	6	14	2.33	Q3	2.7
16	FRONTIERS IN CELL AND DEVELOPMENTAL BIOLOGY	6	2	0.33	Q1	4.6

### 3.5 Analysis of keywords co-occurrence, clusters and bursts

**Figure 11:**
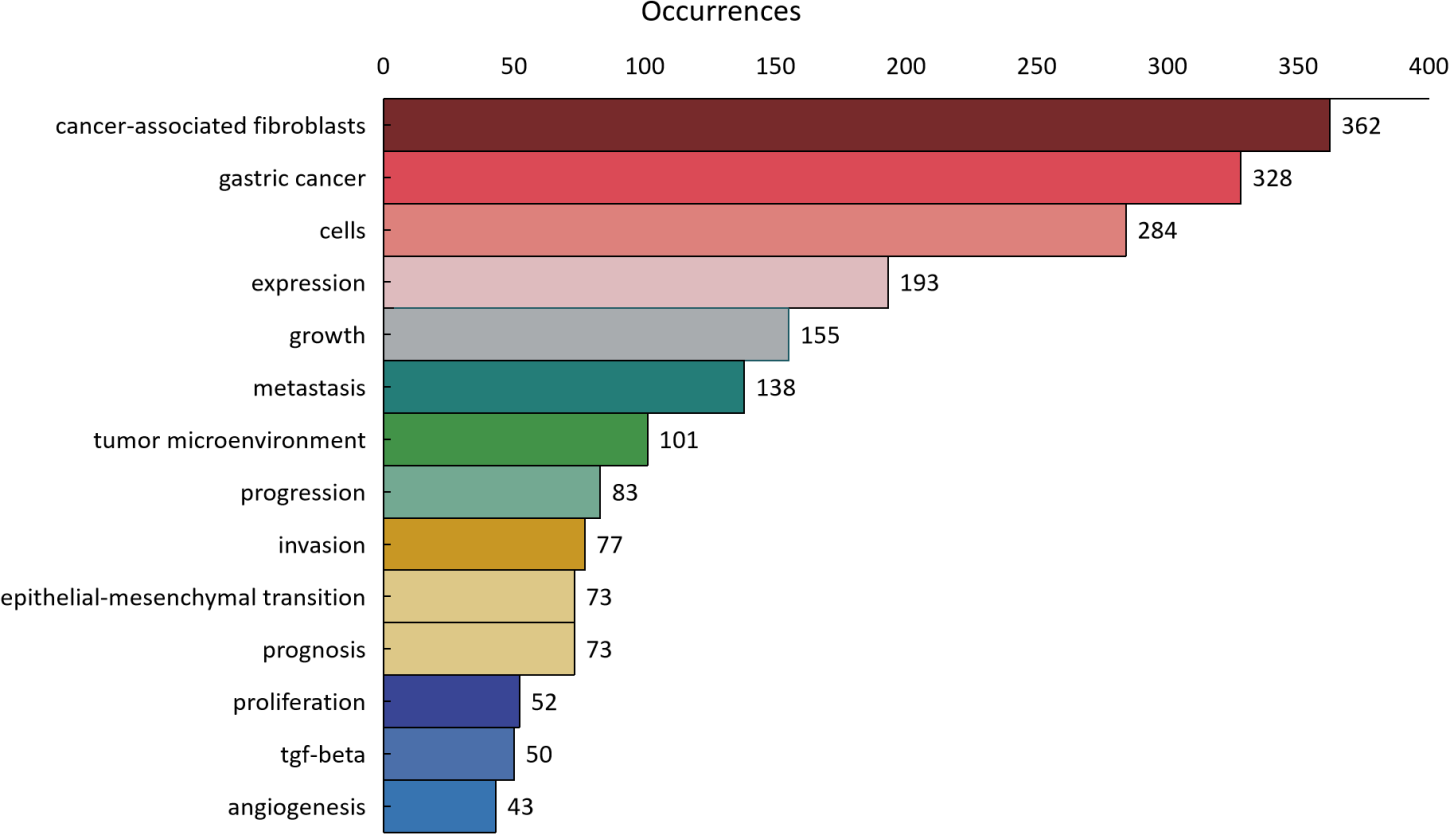
The distribution of keyword occurrence times.

**Figure 12:**
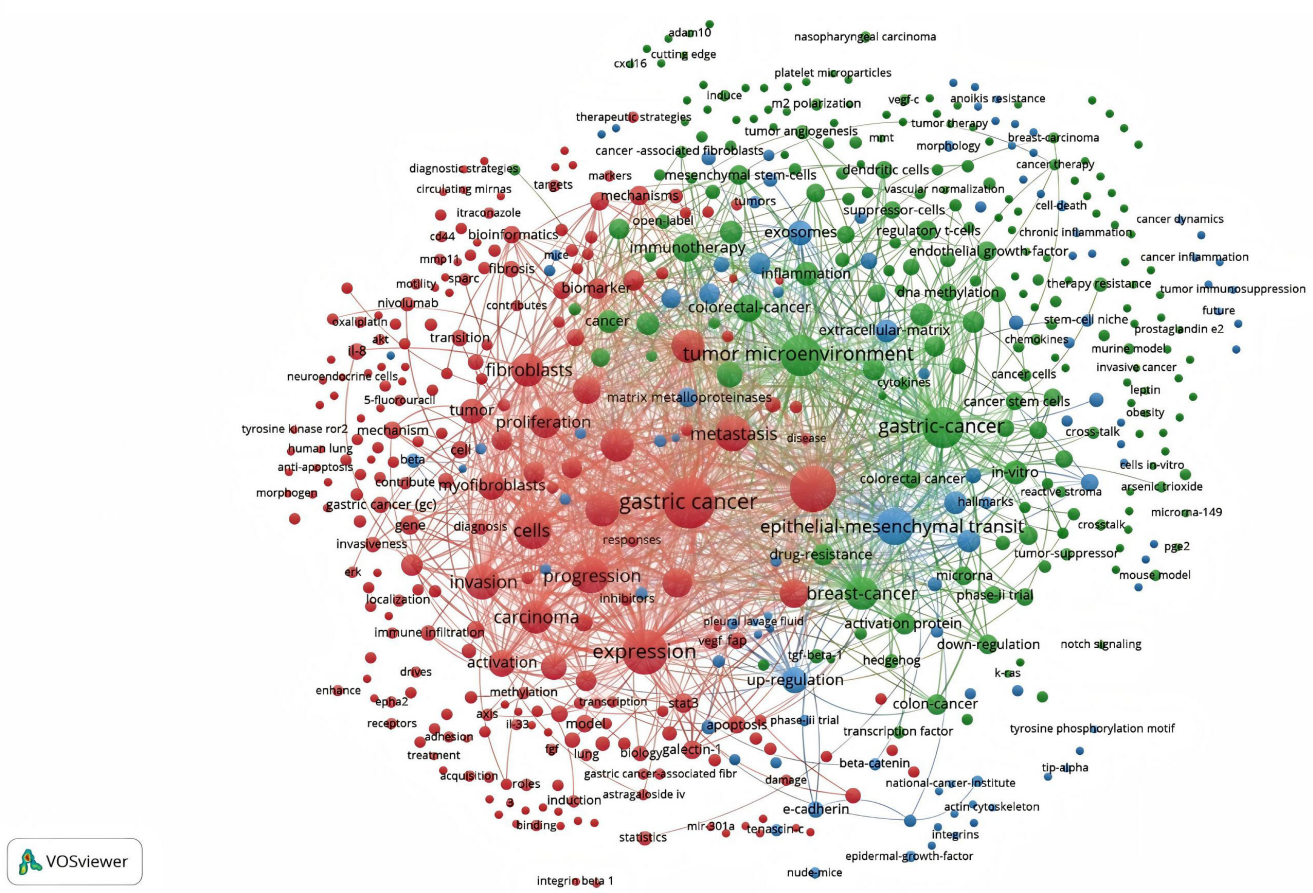
Network map of keywords for cancer-associated fibroblasts and gastric cancer studies.

**Figure 13:**
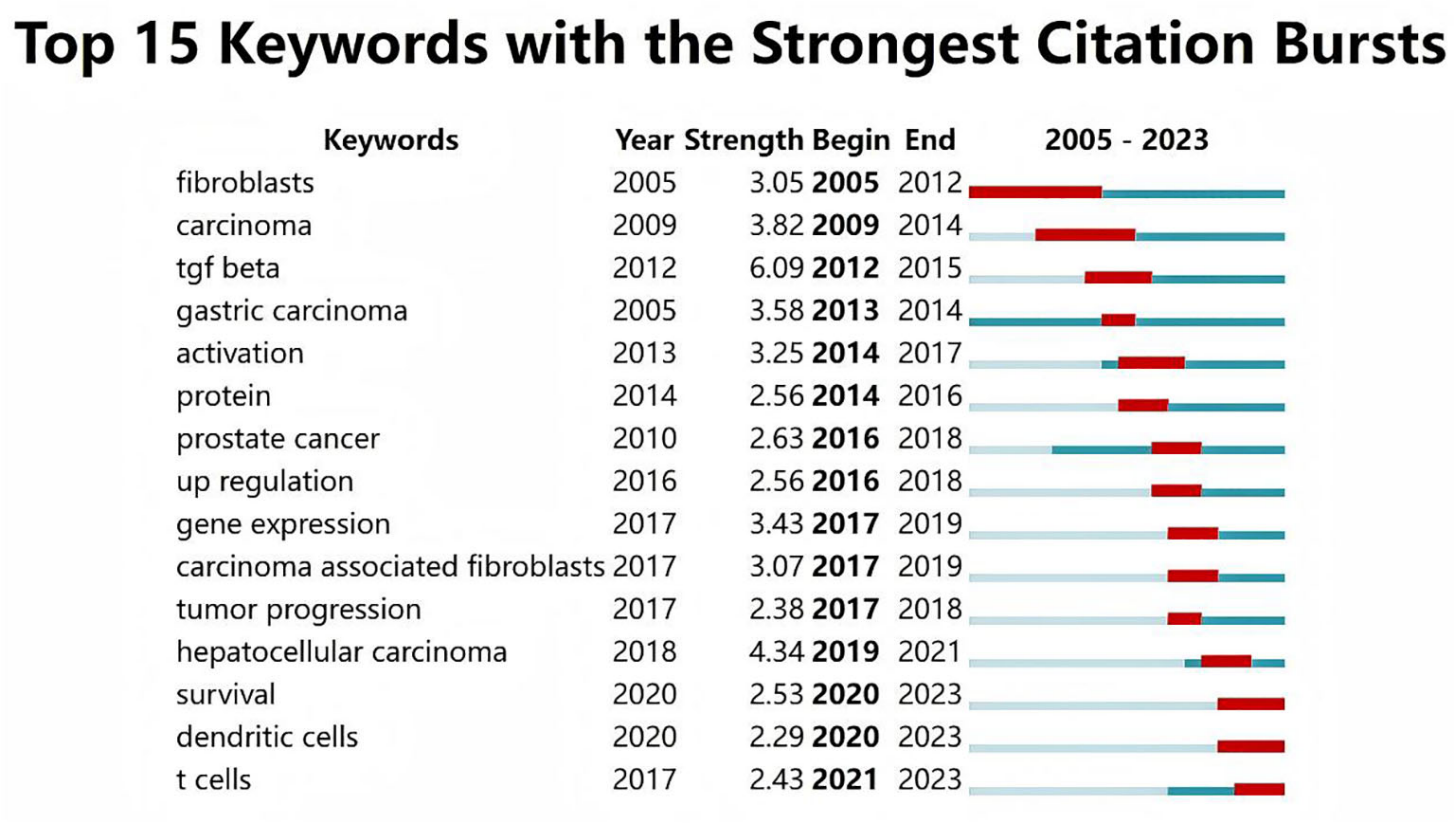
The top 15 keywords with the strongest citation bursts.

As shown in **Figure 11**, 1800 keywords were extracted from the 418 papers. Among these, cells(N=284), expression(N=193), growth(N=155), metastasis(N=138), and tumor microenvironment(N=101) were closely related to CAFs and GC. Keyword cluster analysis elucidates topic distribution within CAFs and GC research, enhancing clarity of specific research areas. **Figure 12** visually depicts the keyword network, identifying three distinct clusters. The primary red cluster centers on CAFs’ roles in tumor pathology, including growth, metastasis, invasion, and progression. The green cluster focuses on immune regulation involving mesenchymal stem cells, regulatory T cells, epithelial growth factor, and TGF-beta1 within the immune microenvironment. The blue cluster shows CAFs in relation to epithelial-mesenchymal transition (EMT) in the GC immune microenvironment, highlighting exosomes, signaling pathways, and non-coding RNAs. Keyword trend analysis offers insights into shifts in keyword prominence and their temporal patterns. As shown in **Figure 13**, the keywords that experienced an explosion in the early period (2005-2015) mainly included fibroblasts, tumors, and tgf-beta. In the middle period (2016-2018), the incidence of keyword burst changed significantly, especially in research related to the pathological process of cancer, including gene upregulation and promotion of tumor progression. From 2019 to 2023, the main focus of this research is related to the impact of cellular regulation on patient prognosis, with special emphasis on T cells and dendritic cells becoming prominent research areas.

### 3.6 The top 10 most-cited articles

In our study, the top 10 most-cited publications on GCand CAF were published between 2011 and 2021 (**Table 3**). The authors of the top three most-cited publications are Kratochwil, Clemens, Quante, Michael, and Zhang, Haiyang, respectively.

**Table 3:** The top 10 mos-cited articles for cancer-associated fibroblasts and gastric cancer studies .

Rank	Title	Total Citation	First Author	Year
1	68Ga-FAPI PET/CT: tracer uptake in 28 different kinds of cancer	893	Kratochwil, Clemens (22)	2019
2	Bone Marrow-Derived Myofibroblasts Contribute to the Mesenchymal Stem Cell Niche and Promote Tumor Growth	816	Quante, Michael (23)	2011
3	CAF secreted miR-522 suppresses ferroptosis and promotes acquired chemo-resistance in gastric cancer	621	Zhang, Haiyang (24)	2020
4	Cyclooxygenase-2 in cancer: A review	499	Goradel, Nasser Hashemi (91)	2019
5	Signaling pathways in cancer-associated fibroblasts and targeted therapy for cancer	302	Wu, Fanglong (90)	2021
6	FGFR inhibitors: Effects on cancer cells, tumor microenvironment and whole-body homeostasis (Review)	293	Katoh, Masaru	2016
7	IL-6 secreted by cancer-associated fibroblasts promotes epithelial-mesenchymal transition and metastasis of gastric cancer via JAK2/STAT3 signaling pathway	249	Wu, Xiongyan (18)	2017
8	Metabolic coupling and the Reverse Warburg Effect in cancer: Implications for novel biomarker and anticancer agent development	236	Wilde, Lindsay	2017
9	Tea Polyphenols Decrease Serum Levels of Prostate-Specific Antigen, Hepatocyte Growth Factor, and Vascular Endothelial Growth Factor in Prostate Cancer Patients and Inhibit Production of Hepatocyte Growth Factor and Vascular Endothelial Growth Factor *In vitro*	183	McLarty, Jerry	2009
10	Tumor microenvironment in gastric cancers	180	Oya, Yukiko	2020

## 4 Discussion

CAFs are the most abundant stromal cells in tumor microenvironment (TME) which can not only directly change the immunosuppressive effect of TME, but also affect the aggregation of immune cells by secreting a large number of factors, thereby inhibiting the function of immune cells and helping tumor cells easier to immune escape (16). Considering the pivotal role of CAFs in GC and the extensive research focus, we performed a bibliometric analysis utilizing publication data from the Web of Science (WOS). This study seeks to elucidate the developmental trends and prominent research directions concerning the association between CAFs and GC. The sustained increase in global publications reflects significant research interest, suggesting substantial potential for ongoing investigation in this domain.

### 4.1 General distribution

An analysis of publications by country/region and institution reveals discrepancies between publication volume and citation impact. Despite China’s lead in the number of publications, its total and average citation counts lag behind Japan. This discrepancy may be attributed to several factors. On the one hand, a significant portion of Chinese research may be concentrated in the descriptive or preliminary exploratory stages, lacking in-depth mechanistic studies and systematic analyses. In contrast, Japanese research may focus more on full-chain studies from molecular mechanisms to clinical applications, thereby achieving greater academic influence. On the other hand, some Chinese studies may employ relatively traditional methods and techniques, lacking innovation. In comparison, Japanese research may demonstrate more breakthroughs in the application of new technologies and the discovery of new targets, thus garnering more citations. Furthermore, Japan’s emphasis on internationalization and high-level academic training in talent cultivation has attracted a large number of outstanding researchers. Although China has made significant progress in talent cultivation, it still has some gaps in internationalization and the training of high-end talents. Additionally, the top 10 contributors in terms of publications are primarily from Asian countries, highlighting a lack of participation from Western nations. This phenomenon may be attributed to several factors. On the one hand, the incidence of gastric cancer is relatively low in Western countries such as Europe and the United States. In contrast, gastric cancer is more prevalent in Asian countries, including China and Japan, where more substantial research investment and in-depth studies are conducted (17). This disparity in disease prevalence has led to relatively lower attention and resource allocation to gastric cancer research in Western countries. On the other hand, the distribution of research resources in Western countries is more fragmented, making it difficult to concentrate efforts on a specific cancer type as seen in China and Japan. Enhancing research quality and fostering collaboration with esteemed international institutions are crucial for expanding academic influence. Notably, Masakazu Yashiro, Kosei Hirakawa, and Zhenggang Zhu have emerged as leading figures in CAF and GC research, demonstrating significant contributions in authorship and collaborative efforts. These respected individuals have demonstrated exceptional leadership with significant contributions in terms of the number of publications, number of citations, etc. They focused on the mechanism by which CAFs promote tumor growth and progression through tumor metabolism. For example, they demonstrated that CAFs enhance the migration and EMT of gastric cancer cells by activating the Janus kinase 2/signal transducer and activator of transcription (JAK2/STAT3) pathway in gastric cancer cells by secreting IL-6. The depletion of IL-6 with a neutralizing antibody or inhibition of the JAK/STAT3 pathway with the specific inhibitor AG490 significantly attenuated these phenotypes in CAF-induced gastric cancer cells (18). The author collaboration map reveals active international cooperation, yet it remains predominantly concentrated within specific countries and institutions. ‘Frontiers in Oncology’ leads in publication volume, while ‘Oncogene’ garners the most citations. Journals excelling in both publication and citation metrics are primarily situated in the JCR Q1 category, underscoring their significant academic influence. Key journals such as “Frontiers in Oncology”, “Gastric Cancer”, “Cancers”, and “BMC Cancer” serve as primary outlets for CAF research dissemination. However, there remains a notable absence of publications in highly influential international journals like “Science”, “Nature”, and “Cell”. To address this, researchers are encouraged to enhance the quality of their work to achieve results with greater international impact, facilitating global discourse and exchange. Keyword co-occurrence and cluster analysis indicate a research shift from understanding the regulatory roles of CAFs in gastric cancer pathology to developing CAF-targeted cancer therapies. CAFs have been implicated in promoting cancer cell growth, metastasis, and invasion, and are closely tied to tumor progression and prognosis. Based on the keyword burst, CAFs have different research directions. The keyword hotspots of CAFs and GC in the early stage mainly suggest that CAFs affect the progression of gastric cancer cells macroscopically. For instance, the human fibroblast activation protein (FAP), a 97-kilodalton cell surface glycoprotein, is specifically expressed within the context of tumor-associated fibroblasts. Fukumoto et al. examined FAP levels in surgically resected gastric cancer using immunohistochemistry and confirmed that 90% of the patients had lymphatic invasion, indicating that CAFs influence the progression of GC (19). In the middle stage, the pathological mechanism by which CAFs affect cancer cells has been studied in more detail. In a seminal study, Yang et al. reported that the expression of miR-106b is significantly elevated in cancer-associated fibroblasts (CAFs) when compared to normal fibroblasts derived from gastric cancer patients. The overexpression of miR-106b in CAFs was found to markedly enhance the migratory and invasive capabilities of gastric cancer cells through the downregulation of the phosphatase and tensin homolog (PTEN). These findings suggest that miR-106b could represent a promising therapeutic target for gastric cancer intervention (20). In the later stage, more attention has been paid to how other surrounding immune cells affect the prognosis of patients with GC. For instance, fibroblasts with tumor-associated characteristics have been shown to suppress the functionality of CD8+ T-cells through the induction of an IL-6 autocrine signaling loop and by engaging with Th17 cells, thereby modulating the tumor microenvironment (21).

### 4.2 The most influential publications

In the field of GC research involving CAFs, the most cited article is the 2019 publication by Kratochwil, Clemens, titled “68Ga-FAPI PET/CT: tracer uptake in 28 different kinds of cancer” (22). This study highlights that positron emission tomography/computed tomography (PET/CT) is a primary imaging modality for cancer diagnosis, staging, and management. However, traditional fluorodeoxyglucose (FDG)-PET/CT has limitations in tumor delineation and identification of metastatic lesions in cancers with high background uptake, inflammation, or low metabolic activity. Fibroblast activation protein (FAP) is specifically expressed in CAFs, and its overexpression is associated with increased local inflammation, suppression of lymphocyte activity, and poor prognosis in epithelial cancers. FAP inhibitors (FAPIs) based on quinoline, as a novel strategy targeting the tumor stroma, have been developed as radiopharmaceuticals. This study demonstrates that FAP-based PET/CT may provide a complementary imaging strategy to FDG-PET/CT, especially in detecting highly fibrotic tumors and metastatic lesions. 68Ga-FAPI uptake varies among different cancer types and shows the advantage of low background uptake in metastatic lesions of the liver and lungs. These results suggest that FAPI-PET/CT has potential applications in metabolically active tissues. Although this study evaluated 68Ga-FAPI uptake across multiple cancer types, it had a relatively small sample size and was retrospective in nature. Therefore, prospective studies in larger and more diverse clinical settings are needed to further validate the clinical utility of FAPI-PET/CT. Future research could explore the application of FAPI-PET/CT in other cancer types and assess its specific clinical applications. Additionally, further optimization of the chemical structure of FAPI tracers could enhance their uptake efficiency and specificity in different cancer types.

The second most cited article is a 2011 study by Quante, Michael et al (23). In this study, the researchers were aware that CAFs in the tumor microenvironment play a crucial role in tumor progression, but their exact origins and mechanisms of action remained unclear. Using mouse models and cell culture experiments, this study elucidated the role of bone marrow-derived myofibroblasts (MFs) in the tumor microenvironment, which support tumor growth and progression through interactions with mesenchymal stem cells (MSCs). Transforming growth factor-beta (TGF-β) and stromal cell-derived factor-1 alpha (SDF-1α) signaling pathways were found to be critical in the differentiation and recruitment of MFs. These findings provide new targets for developing therapeutic strategies targeting the tumor microenvironment. Although this study revealed the mechanisms of bone marrow-derived MFs in the tumor microenvironment, its direct application to human tumors requires further validation. Additionally, the study mainly focused on a gastric cancer model, and the relationships between MFs and MSCs in other tumor types need further investigation. Future research could explore the mechanisms of MFs and MSCs in other tumor types and assess the potential clinical applications of TGF-β and SDF-1α inhibitors. Moreover, further exploration of other functions of MFs and MSCs in the tumor microenvironment, such as immune regulation and angiogenesis, is warranted.

The third most cited article is “CAF secreted miR-522 suppresses ferroptosis and promotes acquired chemoresistance in gastric cancer” by Zhang, Haiyang et al (24). This study recognized that ferroptosis, a novel form of non-apoptotic cell death caused by the accumulation of iron-dependent lipid peroxidation (lipid-ROS), is supported by CAFs through the secretion of various bioactive substances, including exosomes. However, the role of CAFs in regulating cancer cell lipid metabolism and ferroptosis, as well as their potential involvement in chemoresistance, remained unclear. This study demonstrated that CAFs inhibit ALOX15 expression in gastric cancer cells via exosomal miR-522, thereby reducing lipid ROS accumulation, suppressing ferroptosis, and promoting chemoresistance. USP7 and hnRNPA1 were shown to promote miR-522 secretion by deubiquitination and stabilization of hnRNPA1. This research unveiled a new mechanism by which CAFs regulate ferroptosis in gastric cancer and provided a novel target for enhancing chemosensitivity in gastric cancer. Although this study elucidated the role of CAFs in ferroptosis in gastric cancer, its direct application to human gastric cancer requires further validation, as the study was mainly based on *in vitro* cell experiments and nude mouse models. Additionally, while the study focused on the regulatory mechanisms of miR-522 and ALOX15, other potential molecular mechanisms need further exploration. Future research could investigate the mechanisms of CAFs in other tumor types and assess the potential clinical applications of USP7, hnRNPA1, and miR-522. Moreover, further exploration of other functions of CAFs in the tumor microenvironment, such as unlimited proliferation and immune evasion, is needed.

### 4.3 Hotspots and frontiers

**Figure 14:**
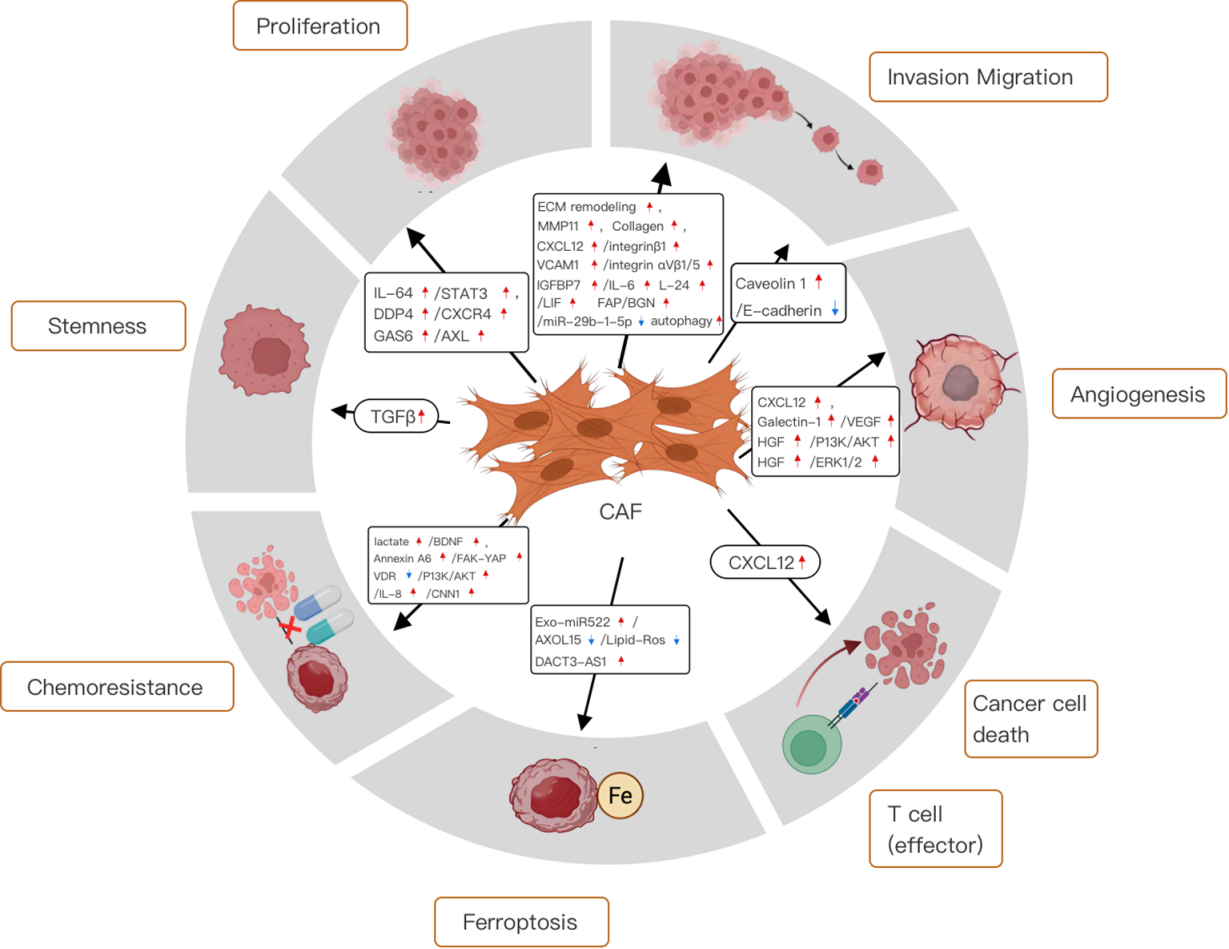
Mechanistic diagram of the different roles of cancer-associated fibroblasts in gastric cancer.

Hotspots and frontiers represent the current status of a research field, which indicates the direction for researchers to explore unknown fields and promote the rapid development of related fields. **Figure 14** summarizes the hot research in the field of CAFs and GC.

#### 4.3.1 Functions of the cancer-associated fibroblasts in the tumor microenvironment

Cancer-associated fibroblasts (CAFs) have unique features in the tumor microenvironment (TME) that distinguish them from normal fibroblasts. More active than their normal counterparts, CAFs exhibit greater proliferation and migration abilities (25). They secrete numerous growth factors and cytokines, including TGF-β, FGF, PDGF, IL-6, and IL-8, which drive cancer cell proliferation (26, 27). When CAFs secrete TGF-β, it binds to specific receptors on cancer cells, activating SMAD proteins. The SMAD complex then functions as a transcription factor in cancer cells, regulating the expression of genes like cyclin D1, BCL-2, and VEGF (28).

CAF also secretes a variety of mediators, such as interleukins, growth factors, and chemokines. These substances not only induce cancer cell proliferation and stemness but also promote epithelial-mesenchymal transition (EMT) and the invasion of surrounding tissues (29). Moreover, by secreting extracellular matrix (ECM) proteins and remodeling enzymes, CAFs alter the ECM, increasing its stiffness (30). This change can impede the diffusion of chemotherapeutic drugs into tumor tissue, leading to chemoresistance. Additionally, CAFs can reshape the cellular milieu of the TME, contributing to an immunosuppressive microenvironment (31).

##### 4.3.1.1 The effects of cancer-associated fibroblasts on non-cellular components of the tumor microenvironment

In healthy tissues, the extracellular matrix (ECM) is in a dynamic equilibrium between continuous degradation and reconstruction, ensuring its structural integrity and normal function. This process involves the synthesis of new matrix proteins to replace the old ones. In most solid tumors, cancer - associated fibroblasts (CAFs) disrupt this balance mainly by over - secreting a variety of ECM proteins. As a result, CAFs cause a significant increase in tissue and matrix stiffness, creating a supportive and favorable environment for tumor growth and invasion (30).

Moreover, CAFs produce large amounts of collagen, the main component of the ECM, leading to the accumulation of collagen fibers within the ECM (29). CAFs also affect the organization and arrangement of collagen fibers within the ECM by secreting enzymes such as lysyl oxidase (LOX), which form covalent bonds between collagen fibers, resulting in cross - linking and a harder and more mechanically deformation - resistant ECM (32). The second major component of the ECM is fibronectin (FN), a glycoprotein involved in cell adhesion, migration, and wound healing (33). Fibronectin produced by CAFs induces the development of fibrosis, characterized by excessive deposition of ECM components and eventually leading to increased tissue stiffness (34).

In many cancers, the level of CAF infiltration and ECM stiffness is associated with tumor aggressiveness (35). The increase in matrix stiffness within the TME significantly impairs drug delivery and uptake in tumor tissues (36). Conventional antitumor drugs usually have a dose-dependent effect and need to reach effective concentrations at the tumor site (37). However, the hardened ECM creates a physical barrier that hinders drug penetration into the tumor. Tumor cells located deeper within the stiff matrix may be more difficult for drugs to reach. In addition, the increased stiffness alters fluid dynamics by increasing interstitial pressure, thereby limiting drug transport from the circulation to tumor tissues. Consequently, the stiff matrix compromises the efficacy of anticancer drugs. For example, doxorubicin is least effective in the breast cancer model with the highest stiffness (37). Paclitaxel and cisplatin also exhibit higher IC50 values in MCF - 7 cells with a stiffer ECM (38).

In addition to establishing physical barriers to impede drug delivery, CAFs also contribute to inducing cellular chemoresistance mechanisms. Cancer models with a stiff matrix coated with fibronectin show enhanced repair of double - strand DNA breaks induced by cytotoxic drugs. This process is controlled by MAP4K4/6/7 kinases, which trigger ubiquitin phosphorylation. Then, phosphorylated ubiquitin induces H2AX recruitment to DNA damage sites, thereby activating DNA repair mechanisms (39). A recent study showed that CAFs can increase the survival rate of gastric cancer cells and reduce cell apoptosis after 5 - fluorouracil (5-FU) treatment. RNA sequencing of CAFs revealed that neuropilin 2 (NRP2), a transmembrane glycoprotein acting as a VEGFR, has the highest transcript level compared to normal fibroblasts (40). The study emphasized that resistance to 5-FU is associated with NRP2 and mediated by stromal - derived growth factor (SDF-1), also known as C-X-C motif chemokine 12 (CXCL12). Silencing NRP2 leads to down - regulation of H2AX and thus DNA damage repair. These findings indicate that CAFs not only support cancer cells through soluble mediators but may also act on receptor proteins to activate survival pathways within cancer cells.

##### 4.3.1.2 The interaction of cancer-associated fibroblasts with immune cells in the tumor microenvironment

Cancer - associated fibroblasts (CAFs) significantly promote tumor progression by interacting with immune cells in the highly interactive tumor microenvironment (TME) and creating an immunosuppressive environment. CAFs can alter both innate and adaptive immune cells, thereby weakening the immune response to tumor cells. Recently, particularly in the context of macrophages and T cells, the interactions between CAFs and immune cells have emerged as crucial regulators of the tumor immunological microenvironment.

###### 4.3.1.2.1 Interaction between cancer-associated fibroblasts and tumor-associated macrophages

Macrophages, part of the innate immune line first of defense, are present in the tumor microenvironment (TME). Here, cancer - associated fibroblasts (CAFs) recruit them via SDF - 1, chemokine ligands (CXCL), and IL - 6 secretion. These macrophages then transform into tumor - associated macrophages (TAMs), amplifying the tumorigenic microenvironment (41). TAMs, in turn, activate CAFs by secreting growth factors like FGF, VEGF, and PDGF, and induce epithelial - mesenchymal transition, potentially generating new CAFs. Critically, CAF - stimulated TAMs suppress cytotoxic cells and promote immunosuppressive regulatory T cells (Tregs) and myeloid - derived suppressor cells (MDSCs), inducing an immunosuppressive TME phenotype (42).

###### 4.3.1.2.2 Interaction between cancer-associated fibroblasts and T cells

CAFs also interact with T cells, key adaptive immune components in TME - mediated antitumor immunity. By suppressing cytotoxic T cells and promoting Tregs, CAFs foster an immunosuppressive milieu, hindering effective immune - mediated cancer cell elimination. Cotoxicyt T cells, especially CD8+ T cells, are crucial for targeting and destroying cancer cells through apoptosis induction. However, CAFs impair these antitumor activities by releasing soluble factors that inhibit cytotoxic T cell function. For instance, CAF - derived TGF - β, IL - 6, and prostaglandin E2 (PGE2) suppress CD8+ T cell proliferation and cytotoxicity, causing T cell exhaustion (43). CAFs inhibit CD8+ T cell proliferation by upregulating PGE2 expression, disrupting cell cycles and downregulating molecules essential for T cell activation and proliferation (44). Beyond directly suppressing CD8+ T cells, CAFs promote Treg generation, recruitment, or activation in the TME via cytokines like TGF - β, IL - 6, and IL - 10. Tregs primarily inhibit cytotoxic T cells to maintain self - tolerance and immune homeostasis (45).

#### 4.3.2 CAFs contribute to GC cell proliferation

One of the hallmarks of cancer is its unlimited proliferative activity, and cancer cells can maintain their proliferative capacity in a variety of forms. An analysis of the relationship between cell expression profiles and clinicopathological features in the TME of 1524 gastric cancer patients showed that the higher the number of CAFs infiltrated in the TME of gastric cancer patients, the worse the clinical prognosis (46). Zhang et al. demonstrated that CAFs can induce STAT3 activation via IL-6 to promote GC cell proliferation (47). In addition, studies have shown that CAF-derived growth arrest-specific 6(GAS6) in gastric cancer can promote the proliferation and migration of gastric cancer cells by phosphorylating receptor tyrosine kinase (AXL). In addition, increased AXL phosphorylation in gastric cancer tissues is associated with decreased overall survival (48). Kushiyama et al. found that CAFs could also mediate cancer cell proliferation by secreting dipeptidyl peptidase-4 (DPP-4) and its receptor C-X-C chemokine receptor 4 (CXCR4), which laid a foundation for the future to delay or even block cancer cell progression through targeted intervention of CAFs (49).

#### 4.3.3 CAFs contribute to GC cell stemness

Cancer-associated fibroblasts (CAFs) have been shown to enhance the stem-like properties of breast cancer cells via the CCL2/NOTCH1 signaling pathway. Additionally, perostin, a key extracellular matrix (ECM) component secreted by fibroblasts, is crucial for sustaining the stemness of breast cancer cells (50, 51). In addition, a recent study has shown that CAFs play an important role in maintaining GC stemness. More specifically, CAF-conditioned medium stimulated spheroid colony formation and upregulated GC stem cell marker expression, whereas GCFβ marker expression was inhibited by a TGFβ inhibitor, indicating that CAF can modulate GC stemness through TGFβ activity (52). Tumor stemness represents the self-renewal ability of cancer cells, and blocking tumor stemness can prevent cancer cells from becoming malignant. This is crucial for controlling tumor progression and needs to be further studied in the future.

#### 4.3.4 CAFs facilitate invasion, migration, and EMT in GC

Metastatic spread is the predominant factor associated with the unfavorable prognosis and reduced survival rates observed in gastric cancer patients, with peritoneal metastasis accounting for approximately 60% of mortality in this patient cohort (53). A plethora of research has delineated that cancer-associated fibroblasts (CAFs) enhance the migratory and invasive properties of gastric cancer cells, either directly or indirectly, through the secretion of growth factors and cytokines. Further studies have shown that CXCL12 derived from CAFs could promote GC cell invasion by promoting the aggregation of integrin β1 on its surface (54). In addition to inducing cytokine release, CAF-mediated TME remodeling promotes tumor invasion and migration. Collagen-rich matrix promoted EMT and GC cell invasion (55). Notably, CAFs may create gaps in the stromal components and basement membranes that are connected by intercellular junctions and mediate the collective migration of cancer cells via MMP-dependent or MMP-independent mechanisms (56, 57). A study on GC showed that CAFs can promote GC migration and metastasis in an MMP-dependent manner (58). In the realm of gastric cancer research, Shen et al. have elucidated the mechanistic role of cancer-associated fibroblasts (CAFs) in enhancing the stem-like characteristics of gastric cancer cells. Specifically, they demonstrated that CAFs secrete vascular cell adhesion molecule 1 (VCAM-1), which engages with the integrin αVβ1/5 on gastric cancer cells, thereby facilitating tumor invasion both *in vitro* and *in vivo*. This interaction suggests that VCAM-1 may serve as a critical mediator in the crosstalk between CAFs and gastric cancer cells, potentially promoting the invasive phenotype of these cells (59). Other studies have found that the crosstalk between tumor cells and CAFs induced by CAFs autophagy can promote cancer progression, and low caveolin-1 (Cav-1, an autophagy marker) in CAFs can predict a shorter survival time of gastric cancer. However, higher levels of microtubule-associated protein 1 light chain 3 B (LC3B), an autophagy marker, are associated with poor invasiveness and longer survival (60). In the context of gastric cancer (GC) research, recent investigations have revealed that insulin-like growth factor binding protein 7 (IGFBP7), secreted by cancer-associated fibroblasts (CAFs), plays a pivotal role in the progression of GC. Specifically, IGFBP7 has been shown to augment tumor-associated macrophage (TAM) infiltration, thereby promoting GC advancement, through the activation of the FGF2/FGFR1/PI3K/AKT signaling axis. Furthermore, the suppression of IGFBP7 expression has been demonstrated to impede GC cell proliferation and invasiveness in both *in vitro* and *in vivo* models, underscoring the potential of IGFBP7 as a therapeutic target in gastric cancer treatment (61). It has also been shown that in diffuse gastric cancer (DGC), IL-1β from mixed polarized macrophages induces the transformation of non-cancerous fibroblasts (NFs) into cancer-associated fibroblast-like (CAF-like) cells. It promotes the malignant phenotype of DGC cells by inducing the secretion of IL-6, IL-24, and leukemia inhibitory factor (LIF) (62). Studies have shown that fibroblast activation protein (FAP), a derivative of cancer-associated fibroblast-like cells (CAFLCs), can activate the JAK2/STAT3 signaling pathway in GC. However, activated STAT3 promotes biglycan (BGN) transcription in GC, generating a BGN/FPE-STAT3 positive feedback loop. This positive feedback loop mediates the interaction between tumor cells and activated mesothelial cells (MCs) to promote peritoneal metastasis (PM) in GC (63). Studies have shown that SLIT2, an axon guidance protein, was recently suggested to be secreted by CAF, which drives gastric cancer cell metastasis by activating NEK9 (64). Wu et al. showed that downregulation of cancer-associated fibroblast exosome-derived miR-29b-1-5p inhibited angiogenesis mimicry and apoptosis, while accelerating gastric cancer cell migration and invasion via the 1/Zonula occluden-1 axis containing the immunoglobulin domain (65). The above studies have clarified the mechanism of cancer cell proliferation, invasion, and EMT, but the mechanism is complex, and further research is needed to identify the key mechanisms and pathways that can truly improve the prognosis of gastric cancer patients.

#### 4.3.5 CAFs control angiogenesis

Pathological angiogenesis is an important feature of cancer. The growth, invasion, and metastasis of gastric cancer are strongly dependent on oxygen and nutrients supplied by blood vessels. CAF promote angiogenesis to maintain malignant tumor proliferation. As mentioned above, CAFs produce CXCL12, which stimulates neovascularization by recruiting bone marrow-derived endothelial progenitor cells *in vivo (* 66). CAF release pro-angiogenic factors such as VEGFA, PDGFC, and FGF2 to stimulate or adversely affect angiogenesis in tumor tissues (67). Galectin-1, a 14 kDa carbohydrate-binding protein with potential proangiogenic effects, is highly expressed in GC CAF and can accelerate angiogenesis in GC by promoting VEGFR2 phosphorylation and VEGF expression (68). In addition, CAFs have been shown that CAFs may directly stimulate tumor angiogenesis via paracrine CXCL12 signaling in GC (69). Ding et al. found that CAF-derived HGF enhanced the proliferation and migration of vascular endothelial cells through the PI3K/AKT and ERK1/2 signaling pathways, and promoted angiogenesis and vasculogenic mimicry in gastric cancer (70). The above studies provide a theoretical basis for blocking the vascular proliferation of cancer cells and need to be further transformed into clinical practice.

#### 4.3.6 CAFs control immunosuppression

The immune system is an important factor affecting the occurrence and development of cancer. Studies have shown that CAFs can inhibit the infiltration of immune cells in a direct or paracrine manner, reduce the recognition and killing of cancer cells by the immune system, and promote cancer progression (71). For example, CAFs can produce CXCL12, thereby inhibiting the anti-GC effects of T cells in the TME (72). Previous studies have also reported that CAF-derived exosome OIP5-AS1 enhances T cell tolerance and immune escape by downregulating miR-142-5p and up-regulating PDL1 (73). The ability to modulate vascular and immune cells ultimately emphasizes the plasticity of CAFs and the possibility of targeting CAFs in antitumor therapy. Some studies have shown that the expression of neuro-oncological ventral antigen 1(NOVA1), a marker of CAFs, is downregulated in the gastric cancer TME, and its downregulation is associated with an increase in M2 macrophages. However, the number of M2 macrophages is closely related to poor prognosis in patients with gastric cancer (74). The surface marker of CAFs, FAP, is also involved in tumor immunosuppression. Studies in animal models have confirmed that FAP+ CAFs can inhibit the antitumor effect of T cells in the microenvironment of gastric cancer and enhance the antitumor effect of immune checkpoint blockers (75). Immunosuppression is protective against cancer cell proliferation and metastasis. Effective blocking of the immunosuppressive effect of CAFs is very important to accurately eliminate cancer cells and avoid metastasis. At present, the mechanism is not clear, which is the focus of future research.

#### 4.3.7 CAFs support GC progression through metabolic changes

As cancer cells progress, CAFs provide many nutrients and undergo different metabolic reprogramming processes, which may be important in promoting tumor progression. Studies have shown that exosomes secreted by CAF are involved in cancer cell metabolism (76). Additionally, CAF-derived miR-522 was recently shown to inhibit ferroptosis-related metabolism in GC (24). Cancer cell metabolism is characterized by unusually active and adaptive energy metabolic pathways to meet the needs for unlimited proliferation and survival. Future research on cancer cell metabolism will focus on an in-depth analysis of its unique metabolic networks and signaling pathways, as well as the development of novel therapeutic strategies targeting these metabolic features to improve the efficacy of cancer treatment and overcome drug resistance.

#### 4.3.8 CAFs promote GC cell chemoresistance

Resistance to chemotherapy remains a serious challenge in the successful treatment of various tumor types. Many studies have shown the response of CAFs to antitumor therapy and their function in chemotherapy resistance (77). Extracellular vesicles from CAF containing annexin A6 have been reported to enhance cisplatin resistance in GC by stabilizing β1 integrin to induce FAK-YAP activation and renal tubular network formation (77). In addition, CAF-derived BDNF promotes the chemoresistance of GC cells to anlotinib through TrkB stimulation. Therefore, blocking the BDNF/TrkB pathway can induce CAFs to overcome anlotinib resistance effectively (78). These observations further support the notion that the CAFs secretome is involved in the regulation of chemoresistance in cancer cells. In a recent investigation, Qu et al. have identified that exosomal DACT3-AS1, originating from cancer-associated fibroblasts (CAFs), functions as a suppressor in gastric cancer (GC) by impeding malignant transformation and oxaliplatin resistance. Specifically, they demonstrated that DACT3-AS1, when delivered to GC cells via CAF-derived exosomes, enhances the sensitivity of these cells to oxaliplatin through the induction of ferroptosis, a form of regulated cell death mediated by SIRT1. This finding suggests that DACT3-AS1 may serve as a potential biomarker and therapeutic target for enhancing the efficacy of oxaliplatin-based chemotherapy in GC (79). Lu et al. showed that high levels of calmodulin 1 expression in gastric CAFs predict poor clinical outcomes in GC patients, and that calmodulin 1 can promote chemotherapy resistance in gastric cancer by increasing matrix stiffness mediated by cancer-associated fibroblasts (80). At the same time, IL-8 has been suggested to be associated with drug resistance in GC patients (81). However, vitamin D receptor activation in cancer-associated fibroblasts can eliminate CAF-derived IL-8 mediated GC oxaliplatin resistance in GC by blocking PI3K/Akt signaling, which provides a promising basis for overcoming GC drug resistance in clinical practice (82). Cancer cell resistance is one of the main causes of treatment failure in patients with gastric cancer. Future research hotspots and directions of drug resistance in cancer cells should explore the mechanism of drug resistance and develop new drugs and treatments that can reverse or overcome drug resistance, especially the identification and targeting of key metabolic enzymes and signaling molecules related to drug resistance in cancer cells, as well as the development of strategies based on nanotechnology, gene editing, and immunotherapy to enhance drug sensitivity.

#### 4.3.9 The mechanism of crosstalk between CAFs and GC cells

**Figure 15:**
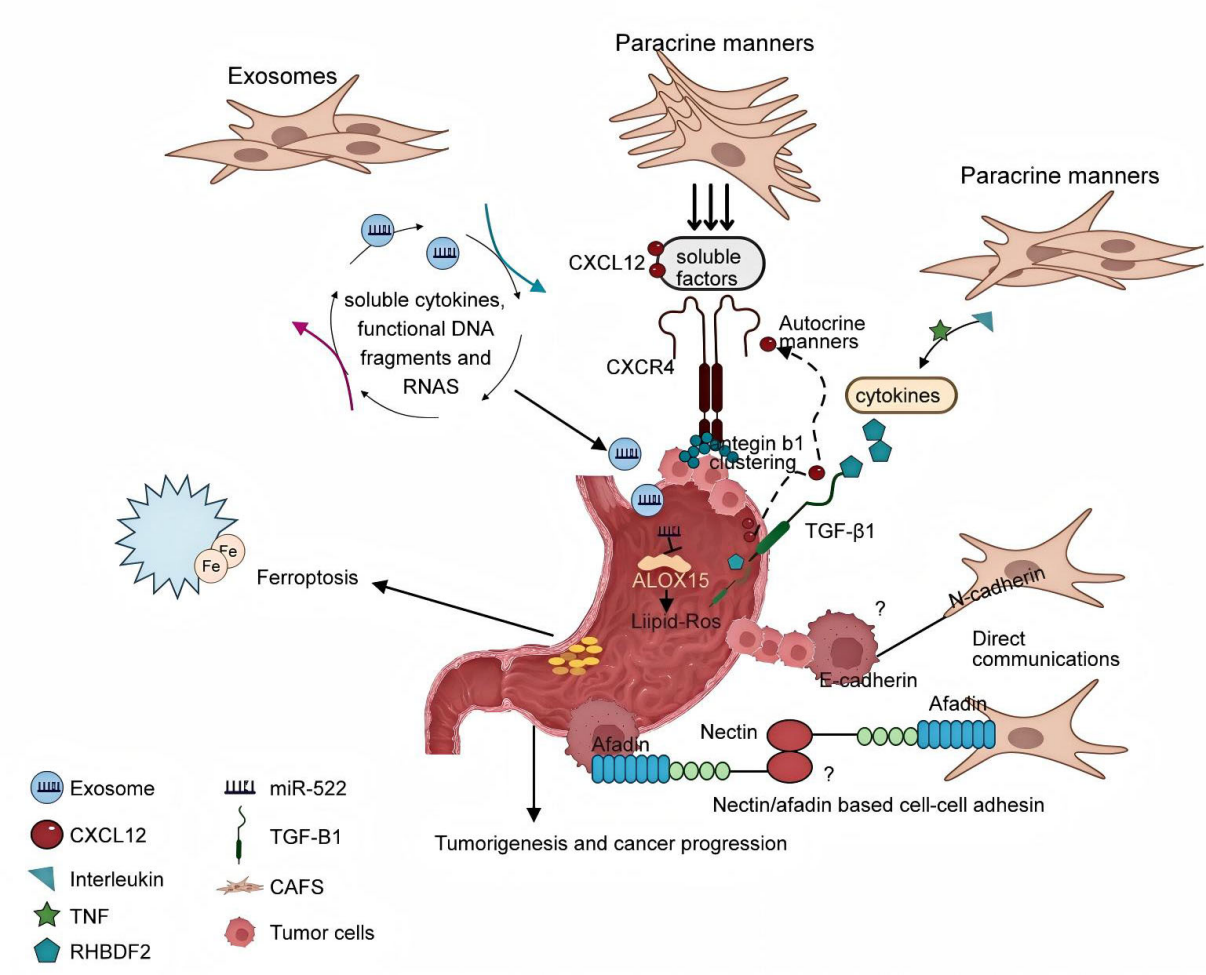
The mechanism of crosstalk interactions between cancer-associated fibroblasts and tumor cells.

Numerous studies have reported that CAFs play an important role in malignant transformation and tumor progression through various behaviors (52, 71), but the mechanism of interaction between tumor cells and CAFs remains to be elucidated (**Figure 15**, CAFs and tumor cells may cooperate to invade through different communication behaviors. One of these communication behaviors may be the chemotactic gradient generated by soluble cytokines to direct cancer cell migration. To a certain extent, CAFs also exhibit heterogeneity in their secretory phenotypes, often showing different patterns of secretion, including paracrine and autocrine signaling. For example, this pro-tumor effect of CAFs can be mediated in GC by CXCL12 and its receptor CXCR4 released by CAF in an autocrine and paracrine manner, respectively (54, 83). Occasionally, cancer cells produce CXCL12; more commonly, they foster an environment involving paracrine signaling and cytokines to stimulate CXCL12 production by stromal cells. Thus, high CXCL12 levels in the TME provide paracrine signaling through a feedback loop that mediates integrin b1 accumulation on the tumor cell surface, promotes tumor EMT, and prevents apoptosis through tumor cell upregulation of CXCR4. Qu et al. have delineated those inflammatory cytokines, including members of the interleukin family and tumor necrosis factor (TNF), secreted by GC cells, upregulate the expression of RHBDF2 in CAFs. This upregulation is linked to the mediation of transforming growth factor-β1 (TGF-β1) signaling, which occurs in a manner independent of the Smad pathway, thereby enhancing the motility of CAFs. Furthermore, this cytokine-mediated interaction promotes the invasive behavior of GC cells through a paracrine mechanism (84). Exosomes, pivotal vectors of intercellular communication, are secreted by tumor and mesenchymal cells and are capable of conveying soluble cytokines, functional DNA fragments, and RNA to mesenchymal cells, thereby augmenting their activation. Notably, exosomes microRNA-522 (miR-522), derived from cancer-associated fibroblasts (CAFs), has been implicated in the acquisition of chemoresistance in gastric cancer (GC) cells by modulating the ALOX15 pathway and impeding lipid-ROS accumulation, as demonstrated both *in vitro* and *in vivo* (52). Exosomes derived from GC cells have been reported to promote the migration and differentiation of umbilical cord mesenchymal stem cells into CAF via the TGF-β/Smad axis (85). Finally, the CAF and tumor cells communicate directly with each other. Labernadie et al. showed that mechanical forces exerted by E-cadherin/N-cadherin heterotypic interactions can coordinate invasion between CAF and tumor cells through two complex mechanisms: CAF may enhance the invasiveness of cancer cells by removing them from the tumor, while cancer cells further promote their spread by guiding CAF migration away from the tumor (86). Connexin acts as an immunoglobulin-like transmembrane cell adhesion molecule that interacts directly with afadin to regulate intercellular adhesion (87). Cancer cells are released from movement restriction owing to the normal contact inhibition provided by the surrounding non-cancer cells expressing liver ligandins. Therefore, elevated livergenin levels in prostate cancer cells may promote local invasion (88). Overall, contact-mediated signaling pathways acting through the Eph/ephrin or nectin/afadin systems may play a crucial role in the crosstalk between cancer cells and CAFs. Despite the direct interaction between CAFs and cancer cells, the glandular architecture of GC tissue maintains the integrity of the basement membrane, thereby blocking its direct connection with stromal cells (84). Until recently, almost nothing was known about direct crosstalk between CAF and GC cells.

#### 4.3.10 Temporal and spatial heterogeneity of CAFs on gastric cancer

Cancer-Associated Fibroblasts (CAFs) display complex biological behavior during gastric cancer progression, with significant temporal and spatial heterogeneity in their roles. Some studies suggest that CAFs may delay cancer onset through unknown mechanisms. The biological function of CAFs in cancer suppression depends on various factors, including the stage of cancer development (89). Several studies have shown that miRNA dysregulation can affect the secretory function of CAFs. Xu et al. found that exosomal miR-139 in gastric cancer is associated with the interaction between CAFs and gastric cancer cells, and miR-139 can suppress gastric cancer progression and metastasis by reducing MMP11 expression in the tumor microenvironment (TME) (58). Notably, Wu et al. pointed out that the inhibitory effects of CAFs may be related to the dynamic balance of the TGF-β signaling pathway. In early gastric cancer, CAFs may secrete TIMP1 to inhibit matrix metalloproteinases (MMPs) and delay tumor invasion. However, in advanced stages, TGF-β may induce epithelial-mesenchymal transition (EMT), shifting CAFs to a pro-cancer phenotype (90). Additionally, Goradel et al. revealed the dual role of the COX-2/PGE2 axis in CAFs. Low-concentration PGE2 may inhibit tumor growth by modulating immune cell activity (91). These findings indicate that CAF functions have temporal and spatial dependence, and their inhibitory effects may exist under specific microenvironment conditions, but further research is needed to clarify the regulatory mechanisms. Overall, the pro-cancer effects of CAFs are far more significant than their anti-tumor effects. Transforming the pro-cancer effects of CAFs into anti-tumor effects is a potential research direction for cancer therapy, but the mechanisms by which CAFs suppress tumors require further study.

#### 4.3.11 Targeted therapy strategies for CAFs

Because the genes of CAFs are more stable than those of cancer cells and are not sensitive to treatment tolerance, it is currently being explored whether changing the subtype, number, or function of CAFs can interfere with the interaction between CAFs and cancer cells, and as a way to improve the treatment effect and prognosis of cancer patients. Currently, the targeting strategies of CAFs can be divided into the following categories: (1) targeting the activation signals of CAFs. TGF-β is an important factor produced by cancer cells that activates tissue-resident fibroblasts into CAFs. TGF-β inhibitors have been used in combination with conventional chemotherapy or immunotherapy to block the pro-tumor signals associated with CAFs in GI tumors (92, 93). (2) CAFs Depletion. Currently, drug delivery systems that target CAFs are being explored. By encapsulating drugs targeting specific surface markers of CAFs with micelles, nanocomposites, and liposomes, CAFs can be killed with a high efficiency and low toxicity. For example, some preclinical trials of FAP antibodies, inhibitors, DNA vaccines, and peptide-drug complexes have shown promising results (94, 95). (3) Targeting CAFs to regulate tumor growth and invasion, angiogenesis, matrix remodeling, and immunosuppression. For example, specific markers of CAFs that exert these functions are directly targeted or related factors secreted by CAFs are targeted to indirectly inhibit their functions (96). (4) restoring CAFs to resting fibroblasts. Studies have found that vitamin A deficiency in pancreatic cancer patients can lead to the activation of pancreatic stellate cells (PSCs); therefore, taking all-trans retinoic acid, an intermediate product of vitamin A metabolism, may restore PSCs to an inactive state (3).

#### 4.3.12 Technical progress in the field of gastric cancer and cancer-associated fibroblasts

Summarizing the technological advancements in the research field of GC and CAFs reveals their critical importance. These advancements enable researchers to gain a deeper understanding of the complex interactions between gastric cancer cells and CAFs within the tumor microenvironment. Moreover, by clarifying these interactions, researchers can pinpoint the key mechanisms that drive tumor progression, metastasis, and therapeutic resistance.

##### 4.3.12.1 Single-cell RNA sequencing technology

Single-cell RNA sequencing (scRNA-seq) has shown great value in the field of gastric cancer and cancer-associated fibroblasts (CAFs) research. This technology can conduct transcriptome analysis on individual cells, effectively deciphering the heterogeneity of CAFs and their subtype-specific expression patterns within the tumor microenvironment. For instance, Kumar et al. used single-cell RNA sequencing to construct a single-cell atlas of gastric cancer, covering 48 samples and identifying 34 different cellular lineage states, including various rare cell clusters. This revealed the specific expression characteristics of CAFs in different tumor subtypes (97). By combining cell surface markers and functional analysis, the technology can further distinguish the origin of CAFs (such as tumor-resident fibroblasts and bone marrow-derived mesenchymal stem cells) and their role in tumor progression (98). For example, it has been found that there is a subpopulation of matrix CAFs in CAFs with high expression of Postn, which promotes the formation of an immunosuppressive microenvironment by activating the Akt signaling pathway in macrophages (99). The advantage of this technology lies in its high-resolution analysis of cellular heterogeneity, but it is costly and has a complex analysis process. In the future, it can be used to further explore the functional changes of CAFs in different stages of gastric cancer and the dynamic interaction mechanisms with tumor cells.

##### 4.3.12.2 Spatial transcriptomics technology

Spatial transcriptomics technology has achieved spatial localization and quantitative analysis of gene expression on tissue sections, providing strong support for studying the distribution of CAFs in gastric cancer tissues and their interactions with tumor cells. For example, Wang R et al. used spatial transcriptomics technology to investigate the expression differences of CAFs in different regions of gastric cancer and found significant differences in gene expression of CAFs between the tumor edge and central regions, revealing their spatial heterogeneity in tumor progression (100). Bae CA et al. used spatial transcriptomics technology to analyze the spatial distribution and interactions of CAFs and other cells in gastric cancer tissues. The results showed that CAFs are closely adjacent to cancer cells in gastric cancer tissues, and the interaction between CAFs and cancer cells promotes the progression of gastric cancer through the secretion of various cytokines and growth factors, such as GAS6 and AXL (48). This technology can intuitively show the neighboring relationship between CAFs and tumor cells and their molecular communication, but its resolution is limited by the thickness of tissue sections and the size of probes. In the future, it is expected to be combined with single-cell RNA sequencing technology to further parse the fine spatial structure and functional zoning of CAFs in the gastric cancer microenvironment.

##### 4.3.12.3 Transcription factor binding site analysis technology

Transcription factor binding site analysis technology plays an important role in elucidating the key gene regulatory mechanisms in CAFs. For example, Grunberg N et al. discovered through chromatin immunoprecipitation (ChIP) technology combined with quantitative PCR that heat shock factor 1 (HSF1) in CAFs regulates the expression of oncogenes by binding to specific gene promoter regions, thereby affecting the malignant phenotype of gastric cancer cells (101). Ma Y et al. found through transcription factor binding site analysis technology that the downregulation of miR-214 in CAFs is related to the invasion and metastasis of gastric cancer. Further studies have shown that miR-214 can inhibit the migration and invasion of gastric cancer cells by directly targeting FGF9 and regulating the EMT process, and its mechanism involves changes in the binding sites of transcription factors with related gene promoter regions, affecting gene expression and thereby influencing the progression of gastric cancer (102). This technology can accurately locate the binding sites of transcription factors with DNA and reveal the molecular basis of gene transcription regulation. However, it relies on high-quality specific antibodies, and the experimental process is cumbersome with significant background signal interference. In the future, it can be jointly applied with other omics technologies to comprehensively parse the transcriptional regulatory network in CAFs and provide potential targets for gastric cancer treatment.

##### 4.3.12.4 Circulating tumor cell detection technology

Circulating tumor cell (CTC) detection technology can monitor the dynamic changes of tumor cells and CAFs during the treatment of gastric cancer. Y Lu et al. analyzed the characteristics and quantity changes of circulating tumor cells in the blood of gastric cancer patients through CTC detection technology and found that the appearance of circulating tumor cells is closely related to the progression, metastasis, and prognosis of gastric cancer. Moreover, the alteration of CAFs in the tumor microenvironment may affect the release and survival of circulating tumor cells, providing evidence for the application of CTC detection technology in the diagnosis and monitoring of gastric cancer (103). Wang R et al. found through CTC detection technology that CAFs-related therapy can significantly reduce the number of CTCs in the peripheral blood of patients, indicating the potential therapeutic effect on inhibiting tumor metastasis (100). This technology has the advantages of being non-invasive and enabling real-time monitoring, but the capture efficiency and detection sensitivity of CTCs need to be improved. In the future, it can be combined with other liquid biopsy technologies to comprehensively evaluate the inhibitory effect of CAFs-related therapy on tumor dissemination.

##### 4.3.12.5 Luciferase reporter gene assay technology

Luciferase reporter gene assay technology has a unique advantage in studying the mechanism by which CAFs-secreted factors affect the function of gastric cancer cells. For example, researchers constructed luciferase reporter genes downstream of specific promoters and assessed the regulatory effect of IL-8 secreted by CAFs on the transcription of key downstream signaling pathway genes in gastric cancer cells by detecting changes in luciferase activity (104). Yang TS et al. confirmed through luciferase reporter gene assay technology that miR-106b can directly target the 3’UTR of the PTEN gene. In gastric cancer cells, the upregulation of miR-106b promotes cell migration and invasion, and the luciferase reporter gene assay technology further clarified the targeting relationship between miR-106b and PTEN, revealing the important role of miR-106b in CAFs in the progression of gastric cancer (20). This technology is highly sensitive and real-time, but it requires ensuring the accuracy of reporter gene construction and cell transfection efficiency. In the future, it can be extended to the study of signaling communication between CAFs and other cell types to uncover new cellular communication mechanisms.

##### 4.3.12.6 Gene editing technology

Gene editing technologies such as CRISPR/Cas9 can be used to construct cell models with specific gene modifications in the study of gastric cancer and CAFs. For example, researchers used this technology to knock out specific miRNAs in CAFs and observed their effect on the proliferation, migration, and invasive ability of gastric cancer cells, thereby clarifying the function of miRNAs in the interaction between CAFs and tumor cells (24). Ma Y et al. used gene editing technology to knock down the SPARC gene in GCAFs. The results showed that GCAFs with SPARC gene knockdown can promote the stemness transformation of gastric cancer cells and their resistance to 5-Fu, and gene editing technology further revealed the involvement of related signaling pathways (such as AKT/mTOR and MEK/ERK signaling pathways) in this process (102). The advantage of gene editing technology lies in its ability to precisely manipulate gene expression, but there is a risk of off-target effects, and it may affect cell activity. In the future, it can be combined with single-cell sequencing technology to further study the transcriptome changes of CAFs after gene editing and their impact on the tumor microenvironment.

#### 4.3.13 Models to study CAFs and GC

The significance of major experimental models in the field of GC and CAFs lies in their ability to not only help researchers gain a deeper understanding of the complex interactions between gastric cancer cells and CAFs within the tumor microenvironment but also accelerate the development and testing of new drugs, promote the exploration of personalized medicine approaches, bridge the translation from basic research to clinical application, and foster interdisciplinary collaboration, thus bringing new hope and strategies for gastric cancer treatment (**Table 4**).

**Table 4:** major experimental models in the field of GC and CAFs.

Experimental Model	Research Use	Advantages	Disadvantages	Future Applicable Research Directions
Cell line model	To study the effects of CAFs on the proliferation, migration, and invasion of gastric cancer cells; to investigate signaling pathways between CAFs and gastric cancer cells	Low cost and easy operation, suitable for high-throughput screening and gene editing; precise control of experimental conditions *in vitro*, facilitating the study of direct CAFs-gastric cancer cell interactions; rapid hypothesis verification, ideal for preliminary experiments	Lack of complexity of the *in vivo* tumor microenvironment, such as extracellular matrix, other stromal cells, and immune cells interactions; cell lines may differ from original tumor cells due to extensive passaging; inability to mimic tumor heterogeneity and dynamic changes	Suitable for preliminary gene function studies and drug screening, especially for studies on CAFs-secreted factors
Tissue sample model	To study the distribution, marker expression of CAFs in gastric cancer tissues, and their spatial relationships with tumor cells	Provides a real tumor tissue environment, preserving the spatial relationships between CAFs, tumor cells, and other stromal cells; allows for studying CAFs characteristics in different pathological types and tumor stages; directly correlates CAFs expression with patient clinical outcomes	Limited sample quantity with significant individual differences and standardization challenges; tissue processing and staining may affect results; difficult to conduct dynamic observations and functional experiments	Suitable for studying CAFs characteristics and prognostic significance in different tumor stages and pathological types
Animal model	To study the role of CAFs in gastric cancer initiation, progression, invasion, and metastasis; to investigate CAFs-immune system interactions	Can simulate *in vivo* tumor growth and metastasis, enabling the study of dynamic CAFs changes in the tumor microenvironment; suitable for evaluating the impact of CAFs on immunotherapy; allows observation of CAFs interactions with the host immune system and other stromal cells	High cost, long experimental periods, and numerous ethical restrictions; differences between animal models and human tumors may affect result extrapolation; high technical requirements for tumor model establishment and manipulation	Suitable for studying dynamic CAFs changes in the tumor microenvironment and their impact on immunotherapy
Organoid model	To study the interactions between CAFs and gastric cancer cells and their effects on tumor cell stemness and chemoresistance	Simulates the tumor’s 3D structure *in vitro*, preserving tumor tissue heterogeneity and cell interactions; suitable for studying CAFs-tumor stem cell interactions; enables high-throughput drug screening to evaluate CAFs’ role in chemoresistance	High technical and cost requirements for organoid culture and maintenance; may not fully replicate the *in vivo* tumor microenvironment; challenges in result reproducibility and standardization	Suitable for studying the mechanisms of CAFs in tumor stem cell maintenance and chemoresistance
Single-cell sequencing model	To study the heterogeneity, subpopulations of CAFs, and their communication networks with tumor cells and other stromal cells	Enables parsing of single-cell gene expression profiles in the tumor microenvironment, revealing CAFs heterogeneity and functional states; identifies different CAFs subpopulations and their roles in the tumor microenvironment; facilitates constructing CAFs molecular maps and cell communication networks	Complex data analysis requiring substantial computational resources and bioinformatics expertise; high experimental costs and limited sample throughput; possible loss of cell spatial information	Suitable for in-depth studies on CAFs subpopulation characteristics and functional diversity in the tumor microenvironment
Clinical sample model	To study the clinical significance of CAFs, including their relationships with patient prognosis, survival rates, and treatment responses	Directly studies CAFs expression and function in patient samples, linking to clinical outcomes; validates CAFs’ clinical value as prognostic markers and therapeutic targets; enables cohort studies to assess CAFs’ roles in different clinical pathways	Complex sample collection and processing with potential ethical issues; significant sample heterogeneity and individual differences making standardization difficult; retrospective studies may have selection biases	Suitable for validating the clinical value of CAFs as prognostic markers and therapeutic targets
Bioinformatics model	To predict key regulatory genes, signaling pathways of CAFs, and potential drug targets	Integrates multi-omics data to predict key CAFs regulatory genes and signaling pathways, guiding experimental design; facilitates mining CAFs-related biomarkers and exploring new therapeutic targets; enables big data analysis to uncover hidden biological patterns	Requires extensive data processing and analysis capabilities with experimental validation needed for prediction results; complex model establishment and optimization with high technical barriers; data quality and completeness may affect analysis outcomes	Suitable for mining CAFs-related biomarkers and exploring new therapeutic targets

Bibliometric analysis serves as a crucial tool for delineating the evolution of research and pinpointing salient domains within the nexus of CAFs and GC. However, this methodology is encumbered by several inherent limitations. Firstly, only English-written articles recorded in the WOSCC database were included. English-language papers are usually in high-impact international journals with strict peer review, ensuring quality and credibility. The WOSCC database covers most high - quality research, so this approach doesn’t affect the overall trend of results. The results of the follow-up literature are detailed in **Supplementary Material S2**. Secondly, only original articles and review articles were included. Original articles represent the latest findings and innovations in the field, while reviews offer a comprehensive framework and background knowledge. Choosing these two types better reflects the field’s full picture and characteristics, making the research more representative and convincing. Their citation patterns also well-reflect the citation network and knowledge flow in the field, providing solid data support for bibliometric citation analysis. Thirdly, during the research, regular checks and corrections of the extracted data were done to ensure its accuracy and completeness. However, due to citation lag, some high-quality recently published studies might not have received enough attention yet and need to be updated in future research. Despite this, this study will greatly assist relevant researchers in understanding the development, hotspots, trends, and frontiers of GC and CAFs, and in identifying areas needing further research.

## 5 Conclusion

In conclusion, the field of CAFs and GC is experiencing rapid expansion, with a growing anticipation that therapies targeting CAFs will emerge as a significant research frontier. Future research endeavors will necessitate extensive international collaboration to yield novel perspectives and chart the course for ongoing investigation and enhancement of CAF-focused strategies.

## Data Availability

The original contributions presented in the study are included in the article/**Supplementary Material**. Further inquiries can be directed to the corresponding author.
