# Supplementary material for: Hotspots and frontiers of the relationship between gastric cancer and cancer-associated fibroblasts: a bibliometric analysis
Source: Front Oncol. 2025 May 23;15:1576696. doi: 10.3389/fonc.2025.1576696 (PMC12140985; doi:10.3389/fonc.2025.1576696)
Supplement: Supplementary file 1 [file DataSheet1.docx]

(TS=("cancer-associated fibroblast*" OR "tumour-associated fibroblast*" OR "tumor-associated fibroblast*" OR "tumor associated fibroblast*" OR "tumour associated fibroblast*" OR "cancer associated fibroblast*" OR "tumor-related fibroblast*" OR "tumor related fibroblast*" OR "carcinoma-associated fibroblast*" OR "carcinoma associated fibroblast*" OR "tumor associate fibroblast*" OR "cancer-associated myofibroblast*" OR "cancer associated myofibroblast*" OR "tumor-associated myofibroblast*" OR "tumor associated myofibroblast*" OR "tumour-associated myofibroblast*" OR "tomour associated myofibroblast*" OR "tumour associate fibroblast*" OR "cancer associate fibroblast*" OR "carcinoma associate fibroblast*" OR "cancer-related fibroblast*" OR "cancer related fibroblast*" OR "tumour-related fibroblast*" OR "tumour related fibroblast*" OR "carcinoma-related fibroblast*" OR "carcinoma related fibroblast*")) AND TS=("gastric cancer" OR "stomach cancer" OR "gastric carcinoma" OR "stomach carcinoma" OR "gastric neoplasm" OR "stomach neoplasm" OR "gastric tumor" OR "stomach tumor" OR "gastric tumorous" OR "stomach tumorous" OR "gastric neoplastic" OR "stomach neoplastic")
